# Chiral Silica with Preferred-Handed Helical Structure via Chiral Transfer

**DOI:** 10.1021/jacsau.1c00098

**Published:** 2021-04-01

**Authors:** Kei Manabe, Sung-Yu Tsai, Satoshi Kuretani, Satoshi Kometani, Katsuyuki Ando, Yoshihiro Agata, Noboru Ohta, Yeo-Wan Chiang, I-Ming Lin, Syuji Fujii, Yoshinobu Nakamura, Yu-Ning Chang, Yuta Nabae, Teruaki Hayakawa, Chien-Lung Wang, Ming-Chia Li, Tomoyasu Hirai

**Affiliations:** † Department of Applied Chemistry, Faculty of Engineering and Graduate School of Engineering, Osaka Institute of Technology, 5-16-1 Omiya, Asahi-ku, Osaka 535-8585, Japan; § Department of Applied Chemistry, National Chiao Tung University, 1001 Ta Hsueh Road, Hsinchu 30010, Taiwan; ‡ Japan Synchrotron Radiation Research Institute, SPring-8, Sayo, Hyogo 679-5198, Japan; ⊥ Department of Materials and Optoelectronic Science, Center for Nanoscience and Nanotechnology, National Sun Yat-Sen University, Kaohsiung 80424, Taiwan; ¶ Department of Biological Science and Technology, National Chiao Tung University, 1001 Ta Hsueh Road, Hsinchu 30010, Taiwan; □ Department of Materials Science and Engineering, School of Materials and Chemical Technology, Tokyo Institute of Technology, 2-12-1-S8-36 Ookayama, Meguro-ku, Tokyo 152-8552, Japan; ○ Department of Biological Science and Technology, Center For Intelligent Drug Systems and Smart Bio-devices (IDS2B), National Yang Ming Chiao Tung University, Hsinchu 30010, Taiwan

**Keywords:** polyhedral oligomeric silsesquioxane (POSS), stereoregularity, chiral transfer, living anionic polymerization, silica

## Abstract

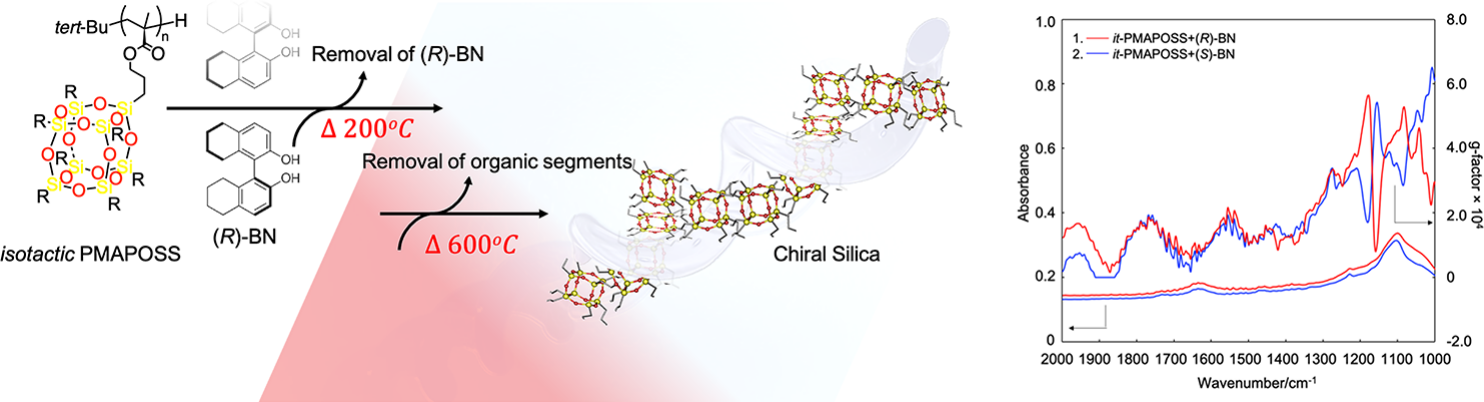

A strategy to obtain chiral silica using an achiral stereoregular polymer with polyhedral oligomeric silsesquioxane (POSS) side chains is described herein. The preferred helical conformation of the POSS-containing polymer could be achieved by mixing isotactic polymethacrylate-functionalized POSS (*it*-PMAPOSS) and a chiral dopant. The array structure of POSS molecules, which are placed along the helical conformation, is memorized even after removing the chiral dopant at high temperatures, leading to a chiral silica compound with exclusive optical activity after calcination.

Silica-based chiral materials such as helical mesoporous silica have attracted considerable attention in the fields of catalysis, templating, and chiral recognition.^1−3^ These materials are prepared by sol–gel transcription using an organic chiral structure as a template.^4−7^ This process is generally tedious, requiring many steps and long reaction times. Moreover, the chemical reaction leads to an aggregated structure approximately few μm to 100 nm in length with a 2–3 nm helical pore. Subnanometer-sized helical structures are difficult to form using sol–gel techniques. In a living organism, polymeric nucleotides such as DNA, RNA, and peptides provide important biofunctional properties through the formation of helical conformations. Helical conformations less than several nanometers in size and with the preferred handedness can simply be formed through noncovalent interactions involving achiral polymers with rigid main chains and chiral dopants.^8^ Numerous polymethacrylate derivatives with inorganic^9^ and metal^10^ precursors have been prepared; however, it is still difficult to form helical structures with the preferred handedness in polymethacrylate derivatives in the absence of complex formation. To the best of our knowledge, preparation of preferred-handed helical silica derivatives using an achiral polymer as a template has not been reported yet.

Polyhedral oligomeric silsesquioxane (POSS) is an organic–inorganic hybrid nanoparticle.^11^ Methacrylate-functionalized POSS (MAPOSS) is commercially available, and its polymers (PMAPOSS) with a well-controlled primary structure were obtained by controlled radical and living anionic polymerization.^9,12,13^ Mesoporous silica^14,15^ could be obtained by performing calcination using a mixture of a block copolymer and POSS derivatives.^16^ A simple and cost-effective preparation method for chiral silica materials can be designed if the helical conformation in PMAPOSS could be controlled and the POSS domains could maintain the helical structure during calcination. As POSS nanoparticles have diameters of about 0.5 nm, it should be possible to form a subnanometer-scale helical structure. Here, we report the fabrication of silica compounds with a helical structure using PMAPOSS preferred-handed helical conformation as the template prepared by mixing isotactic PMAPOSS (*it*-PMAPOSS) and a chiral dopant (Figure 1).

**Figure 1:**
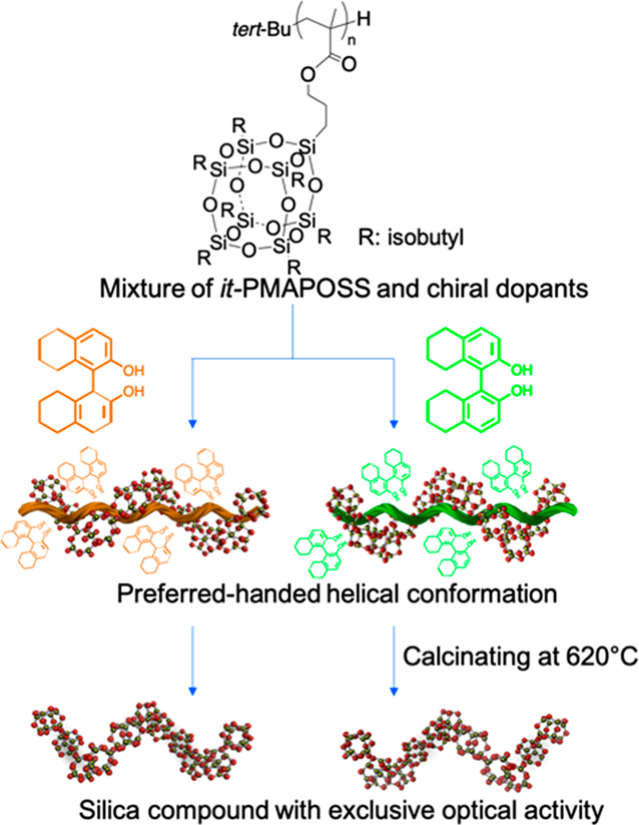
Schematic illustration of the fabrication of a silica compound with the preferred helical structure using *it*-PMAPOSS as the template.

A conventional living anionic polymerization method was used to control the stereoregularity in PMAPOSS (see the Supporting Information and Table S1).^17,18^ Atactic PMAPOSS and isotactic PMAPOSS, designated as *at*-PMAPOSS and *it*-PMAPOSS, respectively, were used. To control the preferred-handed helical conformation of the polymer, *it*-PMAPOSS and (*S*)-(−) or (*R*)*-*(+)-5,5′, 6,6′, 7,7′, 8,8′-octahydro-1,1′-bi-2-naphthol (BN) were mixed and drop-casted on a quartz substrate or silicon wafer. The helical conformation thus induced was evaluated by electronic circular dichroism (ECD) and vibrational circular dichroism (VCD), respectively. Figures 2a and b show the solid-state ECD spectra of *it*-PMAPOSS without and with (*R*)- or (*S*)-BN films, respectively. More importantly, the ECD signals for *it*-PMAPOSS with the enantiomeric BN showed a split-type Cotton effect in the wavelength range of 200–234 nm in the UV absorption spectrum, which can be assigned to the carbonyl group of PMAPOSS. Moreover, the Cotton effect resulted in mirror images of (*R*)- and (*S*)-BN.

**Figure 2:**
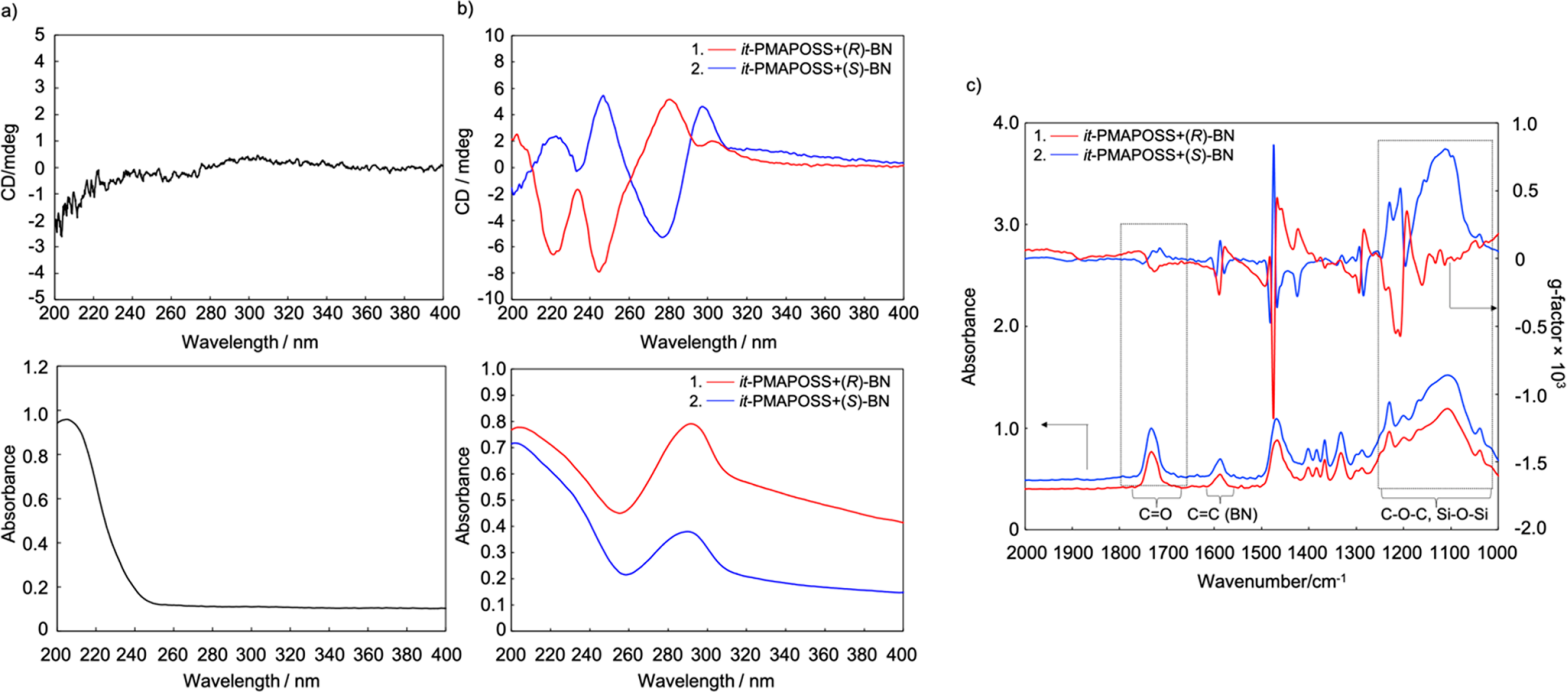
ECD analyses of (a) *it*-PMAPOSS and (b) *it*-PMAPOSS with (*R*)- or (*S*)-BN. (c) VCD spectra of *it*-PMAPOSS with (*R*)- or (*S*)-BN.

To evaluate the effect of stereoregularity on the ECD signals of the carbonyl group, *at*-PMAPOSS with (*R*)- and (*S*)-BN films were also prepared and analyzed by ECD (Figure S7a). No Cotton effect was observed in the wavelength range of 200–234 nm, which implies that stereoregularity strongly affected the molecular aggregation. VCD measurements were performed to further investigate the induced preferred-handed conformation of the helices in PMAPOSS with main-chain chirality. Figures 2c and S7b show the VCD spectra of *it*-PMAPOSS and *at*-PMAPOSS with enantiomeric BN, respectively. Specific split-type and mirror-image VCD signals were observed in *it*-PMAPOSS with enantiomeric BN at ∼1730 cm^–1^ corresponding to carbonyl stretching in the ester group of PMAPOSS. In contrast, split-type VCD signals were not observed at 1730 cm^–1^ for *at*-PMAPOSS with enantiomeric BN. Although several IR peaks ranging from 1000 to 1250 cm^–1^ overlapped, including C–O–C (1050–1250 cm^–1^)^19^ and asymmetric Si–O–Si (1000–1240 cm^–1^)^4^ vibrations, clear split-type Cotton effects could be seen for *it*-PMAPOSS with BN. In contrast to *at*-PMAPOSS with BN, the VCD signals of *it*-PMAPOSS with BN in the range of 1000–1250 cm^–1^ showed mirror-image and split-type Cotton effects and were quite different from those of the (*R*)-BN and (*S*)-BN thin films (Figure S7c) because of the appearance of an induced split-type Cotton effect in the asymmetric Si–O–Si vibrational bands. These results are in good agreement with the ECD results. Thus, we suggest that the split-type Cotton effects of the induced VCD signals in the absorption bands of the C=O, C–O–C, and Si–O–Si vibrations can be attributed to the occurrence of intramolecular interactions with the adjacent POSS pendants twisted in a preferential direction along the polymer main chain, resulting in the induced preferred-handed conformation of the helixes in the *it*-PMAPOSS polymers with main-chain chirality. The morphology of *it*-PMAPOSS with BN films was evaluated by grazing-incidence wide-angle X-ray diffraction (GIWAXD) analysis. The results showed that the blending of the chiral dopant does not affect the thermodynamically stable helix-like structure of *it*-PMAPOSS (see the Figure S16 in Supporting Information).^20^

By taking advantage of the low melting temperature of chiral BN, free chiral BN can be removed by thermal annealing at 200 °C for 30 min. As observed, ECD signals assigned to the carbonyl group of *it*-PMAPOSS are still evident in the 200–234 nm wavelength range (Figure 3a). Next, *it*-PMAPOSS with enantiomeric BN was further annealed at 200 °C for 1 h. During annealing, the intensity of the IR peak at 1584 cm^–1^, which can be assigned to C=C stretching in the phenyl ring of BN, decreased. Finally, the peak vanished at the end of the annealing process (Figure 3b and see the Supporting Information), suggesting that sufficient thermal energy was present to overcome intermolecular hydrogen bonding, leading to the evaporation of enantiomeric BN at this temperature (Figure S10). Most importantly, split-type Cotton effects were evident in the annealed sample at 1730 cm^–1^ and between 1000 and 1250 cm^–1^. It is clear that the preferred-handed helical conformation remained even after BN was eliminated from *it*-PMAPOSS at a high temperature, showing a unique chirality memory effect in stereoregular PMAPOSS. The large steric hindrance associated with the POSS moieties restricts free rotation in the flexible polymethacrylate main chain and further assists in preserving chiral memory.

**Figure 3:**
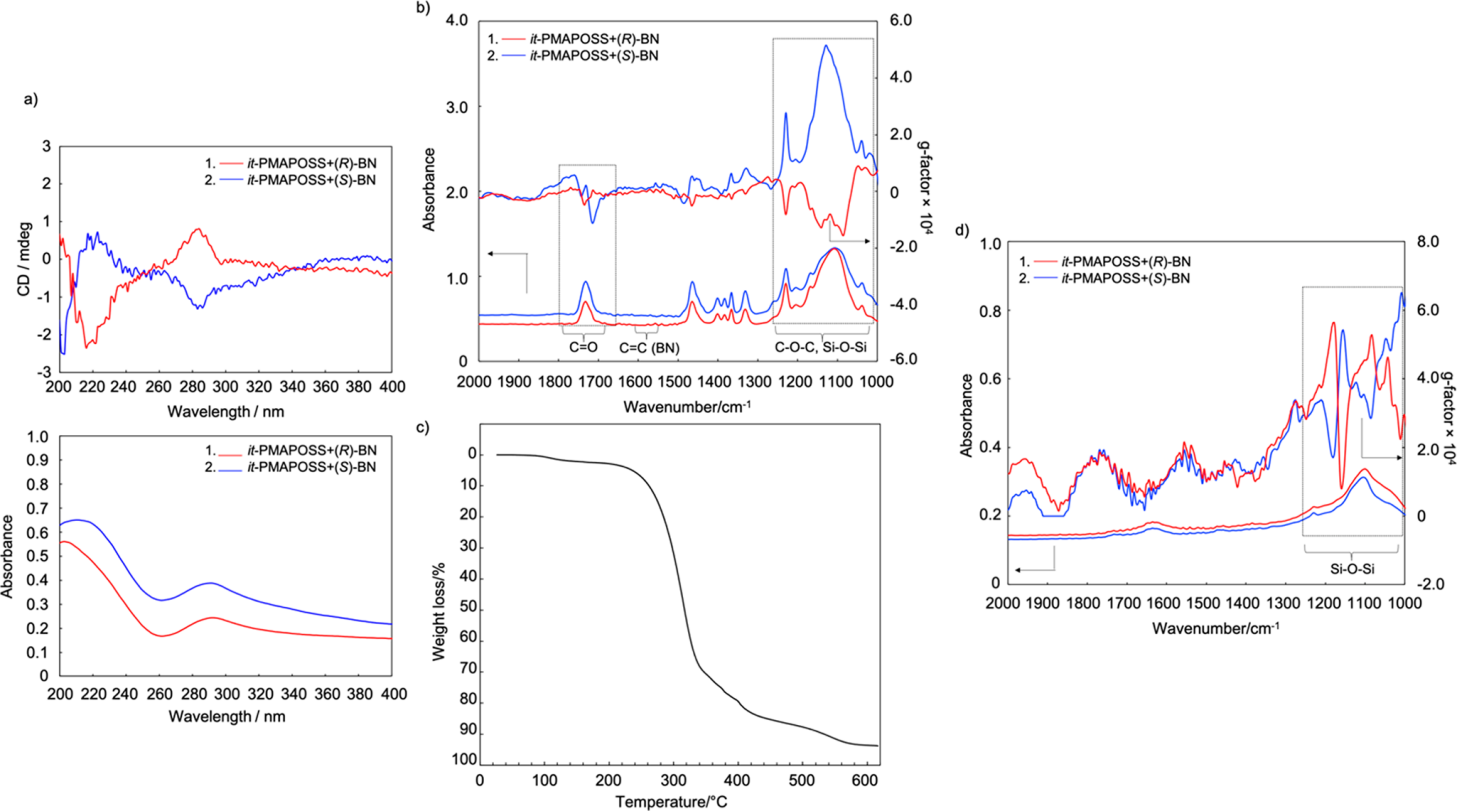
(a) ECD spectra of *it*-PMAPOSS with (*R*)- or (*S*)-BN films annealed at 200 °C for 30 min. (b) VCD spectra of *it*-PMAPOSS with (*R*)- or (*S*)-BN films annealed at 200 °C for 1 h. (c) TGA trace of a mixture of *it*-PMAPOSS and BN. (d) VCD spectra of calcined samples.

Figures 3c and S17 show the thermogravimetric analysis (TGA) measurements for the mixture of PMAPOSS and BN. The measurements were performed from 30 to 620 °C at a heating rate of 10 °C/min under air. The chirality of the residue thus obtained was evaluated using VCD measurements (Figure 3d). The peaks ranging from 1000 to 1240 cm^–1^, which can be assigned to Si–O–Si vibration, were dominant. The two bands centered at 1131 and 1047 cm^–1^ due to the cage and network Si–O bond stretching, respectively, broadened to a single unresolved band, showing a silica structure owing to cage cross-linking.^21^ Hence, it is clear that most of the organic segments were removed from the *it*-PMAPOSS.^16^ Interestingly, the split-type Cotton effect could still be observed in this region and was more striking than that of the sample annealed at 200 °C for 1 h, indicating that the distance between Si–O–Si decreased.

Figure 4 shows the transmission electron microscopy (TEM) image of the calcined sample prepared using a mixture of *it*-PMAPOSS and (*R*)- or (*S*)-BN. A helical structure with the preferred handedness was clearly observed in each calcined sample. The VCD and TEM results suggest that the induced preferred helical structure in the PMAPOSS and BN mixture can be used as a chiral template and maintained during the calcination process, leading to the formation of silica with exclusive optical activity (Figure 1). Hence, this work discusses the preparation of chiral silica using a polymer with well-controlled stereoregularity as a template.

**Figure 4:**
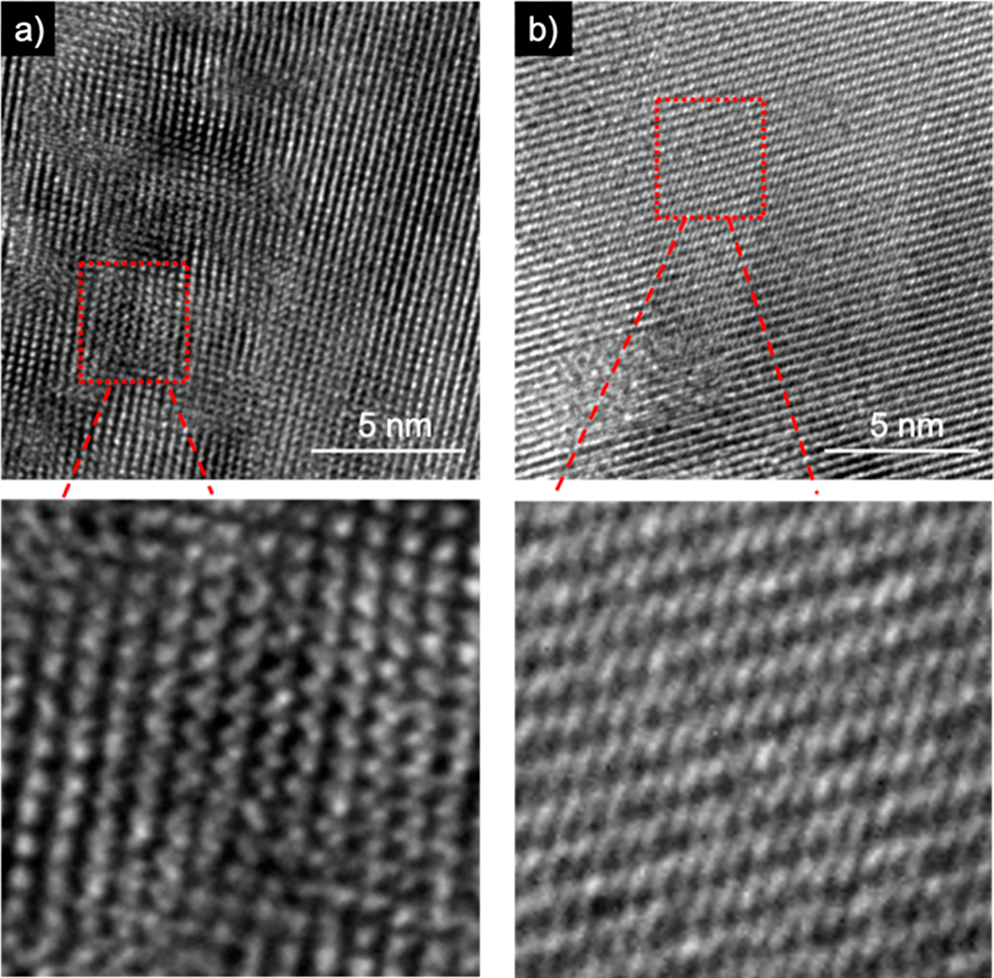
TEM images of calcined samples prepared using a mixture of *it*-PMAPOSS and (a) (*R*)-BN or (b) (*S*)-BN.

In conclusion, *at-* and novel *it*-PMAPOSS were prepared through living anionic polymerization. The *it*-PMAPOSS formed a stable, dynamic, preferred-handed helical conformation, which could be retained even after the evaporation of the enantiomeric BN additives at high temperatures. Moreover, the helical structure was maintained during the calcinating process, leading to the formation of silica with exclusive optical activity. These results are important because they provide an important breakthrough in fabricating chiral silica materials that can be used as a promising material system for applications such as optics, asymmetric catalysts, chiral separation, and medicine.

## Supporting Information Available

Synthesis method, experimental methods, and data analysis (PDF)

## References

[ref1] Rambaud F.; Valle K.; Thibaud S.; Julian-Lopez B.; Sanchez C. One-Pot Synthesis of Functional Helicoidal Hybrid Organic-Inorganic Nanofibers with Periodically Organized Mesoporosity. Adv. Funct. Mater. 2009, 19, 2896–2905. 10.1002/adfm.200900431.

[ref2] Kawasaki T.; Araki Y.; Hatase K.; Suzuki K.; Matsumoto A.; Yokoi T.; Kubota Y.; Tatsumi T.; Soai K. Helical mesoporous silica as an inorganic heterogeneous chiral trigger for asymmetric autocatalysis with amplification of enantiomeric excess. Chem. Commun. 2015, 51, 8742–8744. 10.1039/C5CC01750E. 25803308

[ref3] Che S.; Liu Z.; Ohsuna T.; Sakamoto K.; Terasaki O.; Tatsumi T. Synthesis and characterization of chiral mesoporous silica. Nature 2004, 429, 281–284. 10.1038/nature02529. 15152248

[ref4] Okazaki Y.; Buffeteau T.; Siurdyban E.; Talaga D.; Ryu N.; Yagi R.; Pouget E.; Takafuji M.; Ihara H.; Oda R. Direct Observation of Siloxane Chirality on Twisted and Helical Nanometric Amorphous Silica. Nano Lett. 2016, 16, 6411–6415. 10.1021/acs.nanolett.6b02858. 27585220

[ref5] Jung J. H.; Ono Y.; Hanabusa K.; Shinkai S. Creation of Both Right-Handed and Left-Handed Silica Structures by Sol-Gel Transcription of Organogel Fibers Comprised of Chiral Diaminocyclohexane Derivatives. J. Am. Chem. Soc. 2000, 122, 5008–5009. 10.1021/ja000449s.

[ref6] Cui M.; Zhang W.; Xie L.; Chen L.; Xu L. Chiral mesoporous silica materials: review on synthetic strategies and applications. Molecules 2020, 25, 3899 10.3390/molecules25173899. PMC7504517 32867051

[ref7] Qiu H.; Che S. Chiral mesoporous silica: Chiral construction and imprinting via cooperative self-assembly of amphiphiles and silica precursors. Chem. Soc. Rev. 2011, 40, 1259–1268. 10.1039/C0CS00002G. 21079859

[ref8] Wu Z.-Q.; Nagai K.; Banno M.; Okoshi K.; Onitsuka K.; Yashima E. Enantiomer-Selective and Helix-Sense-Selective Living Block Copolymerization of Isocyanide Enantiomers Initiated by Single-Handed Helical Poly(phenyl isocyanide)s. J. Am. Chem. Soc. 2009, 131, 6708–6718. 10.1021/ja900036n. 19388694

[ref9] Hirai T.; Leolukman M.; Hayakawa T.; Kakimoto M.; Gopalan P. Hierarchical nanostructures of organosilicate nanosheets within self-organized block copolymer films. Macromolecules 2008, 41, 4558–4560. 10.1021/ma800872v.

[ref10] Goseki R.; Hirai T.; Kakimoto M.; Hayakawa T. Iron Oxide Arrays Prepared from Ferrocene- and Silsesquioxane-Containing Block Copolymers. Int. J. Polym. Sci. 2012, 2012, 1 10.1155/2012/692604.

[ref11] Zhang C.; Laine R. M. Hydrosilylation of Allyl Alcohol with [HSiMe2OSiO1.5]8: Octa(3-hydroxypropyldimethylsiloxy)octasilsesquioxane and Its Octamethacrylate Derivative as Potential Precursors to Hybrid Nanocomposites. J. Am. Chem. Soc. 2000, 122, 6979–6988. 10.1021/ja000318r.

[ref12] Pyun J.; Matyjaszewski K.; Wu J.; Kim G.-M.; Chun S. B.; Mather P. T. ABA triblock copolymers containing polyhedral oligomeric silsesquioxane pendant groups: synthesis and unique properties. Polymer 2003, 44, 2739–2750. 10.1016/S0032-3861(03)00027-2.

[ref13] Tsai S.-Y.; Kuretani S.; Manabe K.; Terao T.; Komamura T.; Agata Y.; Ohta N.; Fujii S.; Nakamura Y.; Wang C.-L.; Hayakawa T.; Hirai T. Preparation of polyhedral oligomeric silsesquioxane-containing block copolymer with well-controlled stereoregularity. J. Polym. Sci., Part A: Polym. Chem. 2019, 57, 2181–2189. 10.1002/pola.29498.

[ref14] Coleman N. R. B.; O’Sullivan N.; Ryan K. M.; Crowley T. A.; Morris M. A.; Spalding T. R.; Steytler D. C.; Holmes J. D. Synthesis and Characterization of Dimensionally Ordered Semiconductor Nanowires within Mesoporous Silica. J. Am. Chem. Soc. 2001, 123, 7010–7016. 10.1021/ja015833j. 11459479

[ref15] Beck J. S.; Vartuli J. C.; Roth W. J.; Leonowicz M. E.; Kresge C. T.; Schmitt K. D.; Chu C. T. W.; Olson D. H.; Sheppard E. W.; Et A. A new family of mesoporous molecular sieves prepared with liquid crystal templates. J. Am. Chem. Soc. 1992, 114, 10834–43. 10.1021/ja00053a020.

[ref16] Daga V. K.; Anderson E. R.; Gido S. P.; Watkins J. J. Hydrogen Bond Assisted Assembly of Well-Ordered Polyhedral Oligomeric Silsesquioxane-Block Copolymer Composites. Macromolecules 2011, 44, 6793–6799. 10.1021/ma200926n.

[ref17] Hatada K.; Kitayama T.; Ute K. Stereoregular Polymerization of Alpha-Substituted Acrylates. Prog. Polym. Sci. 1988, 13, 189–276. 10.1016/0079-6700(88)90004-4.

[ref18] Hirai T.; Leolukman M.; Liu C. C.; Han E.; Kim Y. J.; Ishida Y.; Hayakawa T.; Kakimoto M.; Nealey P. F.; Gopalan P. One-Step Direct-Patterning Template Utilizing Self-Assembly of POSS-Containing Block Copolymers. Adv. Mater. 2009, 21, 4334–4338. 10.1002/adma.200900518. 26042939

[ref19] Kawauchi T.; Kumaki J.; Kitaura A.; Okoshi K.; Kusanagi H.; Kobayashi K.; Sugai T.; Shinohara H.; Yashima E. Encapsulation of fullerenes in a helical PMMA cavity leading to a robust processable complex with a macromolecular helicity memory. Angew. Chem., Int. Ed. 2008, 47, 515–519. 10.1002/anie.200703655. 18058787

[ref20] Hirai T.; Leolukman M.; Jin S.; Goseki R.; Ishida Y.; Kakimoto M.; Hayakawa T.; Ree M.; Gopalan P. Hierarchical Self-Assembled Structures from POSS-Containing Block Copolymers Synthesized by Living Anionic Polymerization. Macromolecules 2009, 42, 8835–8843. 10.1021/ma9018944.

[ref21] Fina A.; Tabuani D.; Carniato F.; Frache A.; Boccaleri E.; Camino G. Polyhedral oligomeric silsesquioxanes (POSS) thermal degradation. Thermochim. Acta 2006, 440, 36–42. 10.1016/j.tca.2005.10.006.

