# Supplementary material for: Chiral Silica with Preferred-Handed Helical Structure via Chiral Transfer
Source: JACS Au. 2021 Apr 1;1(4):375–9. doi: 10.1021/jacsau.1c00098 (PMC8395658; doi:10.1021/jacsau.1c00098)
Supplement: Supplementary file 1 — au1c00098_si_001.pdf [file au1c00098_si_001.pdf]

Supporting Information

Chiral Silica with Preferred-Handed Helical Structure via Chiral Transfer

Kei Manabe[†], Sung-Yu Tsai[§], Satoshi Kuretani[†], Satoshi Kometani[†], Katsuyuki Ando[†], Yoshihiro Agata[#], Noboru Ohta[‡], Yeo-Wan Chiang[⊥], I-Ming Lin[⊥], Syuji Fujii[†], Yoshinobu Nakamura[†], Yu-Ning Chang[¶], Yuta Nabae[#], Teruaki Hayakawa[#], Chien-Lung Wang[§], Ming-Chia Li*[,¶,+], and Tomoyasu Hirai*[,†]

[†]Department of Applied Chemistry, Faculty of Engineering and Graduate School of Engineering
Osaka Institute of Technology, 5-16-1 Omiya, Asahi-ku, Osaka 535-8585, Japan

[§]Department of Applied Chemistry, National Chiao Tung University, 1001 Ta Hsueh Road, Hsinchu 30010, Taiwan

[‡]Japan Synchrotron Radiation Research Institute, SPring-8, Sayo, Hyogo 679-5198, Japan

[⊥]Department of Materials and Optoelectronic Science, Center for Nanoscience and Nanotechnology, National Sun Yat-Sen University, Kaohsiung, 80424, Taiwan

[¶]Department of Biological Science and Technology, National Chiao Tung University, 1001 Ta Hsueh Road, Hsinchu 30010, Taiwan

[#]Department of Materials Science and Engineering, School of Materials and Chemical Technology, Tokyo Institute of Technology, 2-12-1-S8-36 Ookayama, Meguro-ku, Tokyo 152-8552, Japan

[+]Department of Biological Science and Technology, Center For Intelligent Drug Systems and Smart Bio-devices (IDS2B), National Yang Ming Chiao Tung University, Hsinchu 30010, Taiwan

Correspondence to: T. Hirai (E-mail: tomoyasu.hirai@oit.ac.jp), and M. -C. Li (E-mail: mingchiali@g2.nctu.edu.tw)

Contents.

1. Materials.

1-1. Instrument. P4-5

1-2. Synthesis of *at*-PMAPOSS and *it*-PMAPOSS. P5-6

1-3. Analysis of interaction between PMAPOSS and BN P6-7

1-4. ECD and VCD spectroscopy. P7-8

1-5. Calcination of *it*-PMAPOSS with BN P8

1-6. GIWAXD measurements P8-9

1-7. Chiral dopant P9

1-8. Effect of polymer chain on the formation of chiral silica P9

1-9. TEM images of calcinated samples prepared by *at*-PMAPOSS with (*R*) or (*S*)-BN. P9-10

2. Supporting Table

Table 1. Primary structure of *at*- and *it*-PMAPOSS P11

3. Supporting Figures. P12-32

Figure S1. SEC curves of *at*-PMAPOSS and *it*-PMAPOSS.

Figure S2. a) ^{1}H and b) ^{13}C NMR spectra of *at*-PMAPOSS and c) ^{1}H and d) ^{13}C NMR spectra of *it*-PMAPOSS.

Figure S3. Highly magnified ^{13}C NMR spectra of *at*-PMAPOSS and *it*-PMAPOSS around a) 45 ppm and b) 176 ppm.

Figure S4. a) Temperature dependence of ^{1}H NMR spectra of *it*-PMAPOSS with (*R*)-BN. b) ^{1}H NMR spectra of (*R*)-BN and *it*-PMAPOSS with (*R*)-BN.

Figure S5. FT-IR spectra of *it*-PMAPOSS (a and b) and *at*-PMAPOSS (c and d) with BN.

Figure S6. ATR-IR spectra of *it*-PMAPOSS (a, b, c, and d) and *at*-PMAPOSS (e, f, and g, and h) with BN.

Figure S7. a) ECD and b) VCD of *at*-PMAPOSS with BN. c) VCD spectra of (*R*) and (*S*)-BN. d) VCD spectra of *it*-PMAPOSS with BN.

Figure S8. ECD and UV-Vis spectra of *it*-PMAPOSS with BN after annealing at 200°C for 30 min.

Figure S9. ^{1}H NMR spectra of *it*-PMAPOSS with BN after annealing at 200°C for 1 h.

Figure S10. Photo images of thermal evaporation of (*R*)-BN and (*S*)-BN in *it*-PMAPOSS with BN. The photo image was taken under 365nm UV irradiation.

Figure S11. ECD spectra of BN film a) CD, b) UV, and c) LD.

Figure S12. a) LD, and b) UV spectrum in ECD measurement for *it*-PMAPOSS film.

Figure S13. a) LD, and b) UV spectrum in ECD measurement for *it*-PMAPOSS with BN.

Figure S14. ECD spectra of *at*-PMAPOSS with BN a) CD, b) UV, and c) LD.

Figure S15. POM images of a) *it*-PMAPOSS and b) *it* -PMAPOSS with BN.

Figure S16. GIWAXD patterns of a) *it*-PMAPOSS and b) *it*-PMAPOSS doped with (*R*)- or (*S*)-BN films. c) Schematic illustrations of the ab and ac projections of the orthorhombic lattice.

Figure S17. TGA and DTA curves for *it*-PMAPOSS with a) (*R*)-BN or b) (*S*)-BN, *at* PMAPOSS with c) (*R*)-BN or d) (*S*)-BN, and e) (*R*)-BN or f) (*S*)-BN monomer.

Figure S18. VCD spectra of *it*-PMAPOSS with 1,1-bi-2-naphthol.

Figure S19. VCD spectra of calcinated sample prepared by MAPOSS monomer with BN.

Figure S20. TEM images of the calcinated samples prepared by *at*-PMAPOSS with a) (*R*) or b) (*S*)-BN.

3. Reference P33

1. Materials.

All solvents and chemicals used in this study were used without further purification except for 3-(3,5,7,9,11,13,15-heptaisobutylpentacyclo [9.5.1^{3,9}.1^{5,15}1^{7,13}]octasiloxan-1-yl)propylmethacrylate (MAPOSS) (Sigma-Aldrich Co. LLC.), toluene (Tokyo Chemical Industry Co., Ltd., 98.0%), tetrahydrofuran (THF) (Sigma-Aldrich Co. LLC., 99.0%), and 1,1-diphenyl ethylene (DPE) (Wako Pure Industries, Ltd., 98.0%). *sec*-Butyllithium (*sec*-BuLi, 1.0 M in cyclohexane) was purchased from Kanto Chemical Industry Co., Ltd. 2-Bromoisobutyl bromide (98%) and methanol (MeOH, 99.5%) were acquired from Sigma-Aldrich. Magnesium turning (99.5%), calcium hydride (CaH_2, 95.0%), lithium chloride (LiCl, 98.0%), diethyl ether (99%), and triethylaluminium ($AlEt_3$, 1.0 M in *n*-hexane) were bought from Wako Pure Chemical Industries, Ltd. 2-Bromo-2-methylpropane (98.0%) and *n*-butyllithium (*n*-BuLi, 1.6 M, *n*-hexane), (*R*)-(+)-5,5',6,6',7,7',8,8'-octahydro-1,1'-bi-2-naphthol ((*R*)-BN, 99.0%), and (*S*)-(-)-5,5',6,6',7,7',8,8'-octahydro-1,1'-bi-2-naphthol ((*S*)-BN, 99.0%) were purchased from Tokyo Chemical Industry Co., Ltd. DPE was distilled from *n*-BuLi. Toluene was distilled from 1,1-diphenyl hexyl lithium. MAPOSS was recrystallized from MeOH and dried under high vacuum condition for 24 h.

1-1. Instrument.

^{1}H (400 MHz) and ^{13}C (100 MHz) nuclear magnetic resonance (NMR) spectra were recorded in $CDCl_3$ or toluene-d_8 using a JEOL JNM-ECZ400S instrument. M_n and PDI were determined by size exclusion chromatography (SEC) using an Agilent 1260 Infinity II (Agilent) instrument with three columns (Tosoh TSKgel Super H2500, TSKgel Super H4000, and TSKgel Super H6000) and 1260 RI detector. THF was used as the eluent with a flow rate of 0.5 mL min^{-1}, and the measurements were performed at 40°C. CD and VCD measurements were performed using J-1700 (CD) (JASCO) and VFT4000 (VCD) (JASCO), respectively. Thermogravimetric analysis (TGA) measurement was conducted using a PerkinElmer Pyris 1. The measurements were performed at air condition and the heating rate was set to 10°C per min. Transmission electron microscope (TEM) measurements were performed using JEOL JEM-2010F and JEM-2100 operated at accelerating voltage 200 kV. The samples were suspended in methanol with ultrasonic vibration for 15 min. The solution was dropped onto carbon coated copper grids for TEM observation. The samples measurements were performed without staining.

1-2. Synthesis of *at*-PMAPOSS[1] and *it*-PMAPOSS.

at-PMAPOSS was prepared by following literature.[1] *it*-PMAPOSS was prepared as following method: 54 mL of diethyl ether and 3.3 g (135 mmol) of magnesium turning were placed in a three-neck flask with a dropping funnel. 12.3 g (90.0 mmol) of 2-bromo-2-methylpropane was diluted by 27 mL of diethyl ether and stocked in the dropping funnel. The mixture was slowly added to the three-neck flask under Ar atmosphere. The solution was stirred for 12 h. The obtained Grignard reagent was stored in Schlenk flask at 0°C until use.

7 mL of toluene was added to a Schlenk flask, subsequently, 0.25 mL of the Grignard reagent was added at -78°C. 1.00 g (1.06 mmol) of MAPOSS was placed in another Schlenk flask and was diluted by 3 mL of toluene. Then, the MAPOSS solution was added to a reaction flask via cannula with vigorous stirring. The polymerization reaction was kept at -78°C for 120 h, subsequently, MeOH was added to quench the reaction. The mixture was precipitated into MeOH. *it*-PMAPOSS (0.82 g) was obtained. Yield: 82%. M_n and PDI were 31,300 and 1.16, respectively. ^{1}H NMR (400 MHz, $CDCl_3$, δ, ppm): 3.74 (s, -OCH_2-), 2,11 (br, -CH_2-, main chain), 1.97-1.75 (br,-CH_2, main chain, C*H,* isobutyl in PMAPOSS), 1.65 (br, -CH_2-, in ester side chain), 1.16 (br, α-CH_3), 1.05-0.86 (be, CH_3 isobutyl in PMAPOSS), 0.68-0.50 (br, Si-CH_2-). ^{13}C NMR (75 MHz, $CDCl_3$, δ, ppm): 175.5, 66.7, 52.5, 45.7, 25.7, 23.8, 21.7, 8.9. Figure S1 shows the SEC curves of *at*-PMAPOSS and *it*-PMAPOSS.

Generally, stereoregularity in polymethacrylate derivatives can be identified using the three ^{1}H NMR signals corresponding to α-methyl groups in the range from 0.8 to 1.2 ppm. These signals can be assigned to *meso-meso* (*mm*), *meso-racemo* (*mr*), and *racemo-racemo* (*rr*) triads from the low to high magnetic field.[2]

Figure S2a and S2c show ^{1}H NMR spectra of *at*-$PMAPOSS_{16}$ and *it*-$PMAPOSS_{33}$. A small peak at 1.2 ppm could be observed in the spectrum of *it*-$PMAPOSS_{33}$ but not in the spectrum of *at*-$PMAPOSS_{16}$. However, other peaks corresponding to *mr* and *rr* overlapped with isobutyl groups on POSS cage, the stereoregularity of PMAPOSS could not be evaluated directly using ^{1}H NMR. Previously, the stereoregularity of polymethacrylate derivatives was evaluated using three signals around 45 ppm in the ^{13}C NMR spectra, which can be assigned to the quaternary carbon next to an α-methyl group.[3] These signals were assigned in the order of *mm*, *mr*, and *rr* from the low to high

magnetic field. *it*-$PMAPOSS_{33}$ showed a strong signal at 45.7 ppm and a weak signal at 45.2 ppm, while *at*-$PMAPOSS_{16}$ showed a single and shoulder at 45.2 and 44.8 ppm, respectively (Figure S3a). To confirm whether *it*-$PMAPOSS_{33}$ included a high *mm* triad, the carbonyl region in its ^{13}C NMR spectrum was evaluated, as shown in Figure S3b. It is widely accepted that *mm* triad could be seen in the higher magnetic region in the carbonyl signals of polymethacrylate derivatives.[3-4] In the case of *it*-$PMAPOSS_{33}$, the single signal at 175.5 ppm could be seen, while three signals were observed at 177.7, 176.8, and 176.3 ppm in *at*-$PMAPOSS_{16}$. Hence, the signal at 175.5 ppm in *it*-$PMAPOSS_{33}$ can be assigned to *mm* triad. This result was in good accordance with the assignment using quaternary carbon in ^{13}C NMR spectrum. The primary structure of *at*-PMAPOSS and *it*-PMAPOSS were summarized in Table S1.

1-3. Analysis of interaction between PMAPOSS and BN.

Figure S4a illustrates the temperature dependence of the ^{1}H NMR spectra of *it*-$PMAPOSS_{33}$ with BN in toluene. Signals corresponding to the OH groups in BN shifted to higher magnetic fields when BN was mixed with *it*-$PMAPOSS_{33}$ (Figure S4b). This is because the hydrogen bonds in BN dissociated and started to associate with *it*-$PMAPOSS_{33}$. The OH signal shifted to higher magnetic fields during the heating process. Moreover, the original single peak converted into bimodal peaks. This implies that the association between *it*-$PMAPOSS_{33}$ and BN dissociated at high temperatures. To understand the interaction between *it*-$PMAPOSS_{33}$ and BN, Fourier-transform infrared (FT-IR) spectroscopy was conducted. As no significant peak shifts could be observed in the IR spectra of mixtures with *it*-$PMAPOSS_{33}$ and BN ratio of 2.27, we mixed them at a ratio of 0.1. Figure S5a shows the IR spectra of *it*-$PMAPOSS_{33}$ and *it*-$PMAPOSS_{33}$ with (*R*)-BN. The peak at 1730 cm^{-1} in the IR spectra can be assigned to C=O stretching vibrations in *it*-PMAPOSS. The peak showed a small shoulder peak in the spectrum of the *it*-$PMAPOSS_{33}$ and (*R*)-BN mixture. This kind of phenomenon could be seen in IR spectra of *it*-$PMAPOSS_{33}$ with (*S*)-BN, *at*-$PMAPOSS_{16}$ with (*R*)-BN, and *at*-$PMAPOSS_{16}$ with (*S*)-BN (Figure S5). To evaluate more detail, the interaction between PMAPOSS and BN was also investigated using attenuated total reflection IR (ATR-IR) measurements. The Si-O in PMAPOSS and -OH vibrational in BN peaks also shift to high wavenumber for *it*-PMAPOSS associated with (*R*)-BN or (*S*)-BN. Similar results can be obtained for *at*-PMAPOSS associated with (*R*)-BN or (*S*)-BN (Figure S6). These

results suggest that the chiral BN associated with not only carbonyl group but also POSS moieties in PMAPOSS. Finally, from the NMR and IR results, it is clear that *it*-PMAPOSS associated with BN via hydrogen bonding.

1-4. ECD and VCD spectroscopy.

All samples for ECD measurements were prepared from the mixture of polymer and BN at the molar ratio of MAPOSS/BN = 2.27 in toluene and the polymer concentration in the solution was maintained at 5wt.%. The solution was annealed at 90°C for 2h, and casted onto a quartz at 90°C. On the other hand, for VCD measurements, samples were prepared as following: PMAPOSS and enantiomeric BN were mixed in toluene at a MAPOSS/BN molar ratio of 2.27 and annealed at 90 °C for 2 h. The solution was then drop-casted on a silicon wafer at 90 °C. Figure S7 shows the a) ECD and b) VCD analysis of *at*-$PMAPOSS_{16}$ with (*R*) and (*S*)-BN. Figure S7c denotes the VCD spectra of (*R*) and (*S*)-BN in film state. Also, *it*-$PMAPOSS_{23}$ with (*R*) or (*S*)-BN, which was described in the main text, was put as Figure S7d.

The intensity of UV peaks in ECD measurement, which are corresponding to the BN in the *it*-$PMAPOSS_{33}$ with BN film decreased after annealing at 200°C for 30 min (Figure S8). This indicates that BN partially evaporated at this temperature. Moreover, this phenomenon is in good accordance with VCD results in Figures 2c and 3b in the main text. The phenomenon was also evaluated using the ^{1}H NMR spectrum. Figure S9 shows the ^{1}H NMR spectra of *it*-$PMAPOSS_{33}$ with BN after annealing at 200°C for 1 h. The signals corresponding to BN can’t be observed. Figure S10 shows the photo image of thermal evaporation of (*R*)-BN and (*R*)-BN in *it*-$PMAPOSS_{33}$ with BN. The photo image was taken under 365 nm UV irradiation. These results strongly indicate that BN is evaporated at this annealing condition.

The linear birefringence (LD) signals affect the ECD signals. The LD signals of each sample were shown in Figures S11-S14 and the signals were small enough. Hence, obtained ECD signals are reliable. Figure S15 shows the polarized optical microscope (POM) images of *it*-$PMAPOSS_{33}$ and *it*-$PMAPOSS_{33}$ with (*S*) or (*R*)-BN film. No optical texture could be seen in the POM images, which indicates that we can neglect the effect of linear birefringence in ECD spectrum. The *it*-$PMAPOSS_{33}$ and *at*-$PMAPOSS_{16}$ in Table S1 were used for ECD measurements, while *it*-$PMAPOSS_{23}$ was used for VCD

measurements.

1-5. Calcination of *it*-PMAPOSS with BN

it-$PMAPOSS_{40}$ and BN were mixed at a molar ratio of MAPOSS/BN = 0.1 in toluene and the solution was annealed at 90°C for 2 h. The solution was casted on the silicon wafer at 90°C. The films thus obtained were scratched and collected. The film was placed in a thermogravimetric analysis (TGA) instrument. The TGA measurement was performed at ranging from 30 to 620°C with heating rate of 10°C per min. The residue was collected and mixed with KBr. The KBr disk including char was evaluated based on the VCD measurements.

1-6. GIWAXD measurements.

GIWAXD measurements were performed on the BL40B2 beamline at the SPring-8 facility in Hyogo, Japan using an incident X-ray wavelength (λ) of 0.1 nm and an incidence angle of 0.16°. The diffraction was detected using PILATUS3 2M (253. 7 mm × 288.8 mm with a pixel size of 172 μm × 172 μm) and Eiger2 500K-BL40B2 (77.3 mm×38.6 mm with 75 μm × 75 μm). The distance between the sample to the detector was calibrated with silver behenate standard and fixed at 337 mm in the case of PILATUS 2M. Whereas the distance of the sample to Eiger2 500K was calibrated with cerium oxide and fixed at 103 mm. The $PMAPOSS_{40}$ was used for this experiment (Table S1).

The morphology of *it*-$PMAPOSS_{40}$ with BN films was evaluated using GIWAXD analysis. Previously we reported that *at*-PMAPOSS film forms orthorhombic crystalline unite cell with dimensions of a = 2.4 nm and b = 5.0 nm and concluded that *at*-PMAPOSS forms helix-like structure to reduce steric hindrance of POSS units.[5] Figure S15a shows the *it*-$PMAPOSS_{40}$ thin film without BN annealed at 220 ℃ for 48 h. The diffraction spots can be assigned by assuming orthorhombic crystallin unite cell with dimensions of a = 2.4 and b = 5.0 nm. The *it*-$PMAPOSS_{40}$ also formed a helix-like structure in the thermodynamically stable state. Figure S16b shows the GIWAXD result for *it*-$PMAPOSS_{40}$ with BN film. The diffraction arcs at higher 2 theta degree could be attributed to the BN and the blending of BN affected the ordered packing of the polymer chain which is the reason why the wide diffraction rings emerged at the pattern. However, two clear diffraction rings at $2\theta_f$ = 1.15°(d_{01} = 5.0 nm) and 2.39° (d_{10} = 2.4 nm) can be observed, which is in good accordance with the size of orthorhombic lattice obtained from

annealed *it*-PMAPOSS$_{40}$. This result confirms that the blending of chiral dopant will not affect the thermodynamically stable helix-like structure of *it* -PMAPOSS. Taking this and CD and/or VCD results into accounts, *it*-PMAPOSS$_{40}$ with BN forms preferred-handed helical conformation and the orthorhombic lattices randomly oriented in the film (Figure S16c).

1-7. Chiral dopant.

To prepare preferred-handed helical conformation in *it*-PMAPOSS$_{40}$, we tried to use several kinds of chiral dopants such as 1-phneylethylamine, BN, and 1,1-bi-2-naphthol. Among them, BN and 1,1-bi-2-naphthol can induce helical conformation in *it*-PMAPOSS, while 1-phneylethylamine, 1-phenylethyl alcohol, and 1-phenyl-2,2,2-trifluoroethanol are not the case. This indicates that chiral dopant with single phenyl ring is not enough to induce the helical conformation in PMAPOSS. This suggests that π-π intermolecular interaction between chiral dopants is essential to induce helical conformation in PMAPOSS. Both PMAPOSS with BN and PMAPOSS with 1,1-bi-2-naphthol samples showed similar VCD signals, which implies that they are forming the same preferred-handed helical conformation (Figure S18).

1-8. Effect of polymer chain on the formation of chiral silica.

To investigate necessity of polymer chain, we mixed MAPOSS monomer and (*R*)- or (*S*)-BN and subsequently calcinated. The silica thus obtained was evaluated using VCD measurements (Figure S19). The VCD signals showed a weak Cotton effect at the ranging from 1000 to 1250 cm^{-1}. This result strongly suggests that polymer chain is necessary and chirality memory effect during annealing process is essential to form chiral silica.

1-9. TEM images of calcinated samples prepared by *at*-PMAPOSS with (*R*) or (*S*)-BN.

TEM images of *at*-PMAPOSS$_{16}$ with (*R*) or (*S*)-BN were shown in Figure S20. The TEM images showed helix-like structure. This might be caused by huge steric hindrance POSS moiety and the main chain in the polymer formed helix like structure. The helix-like structure was already characterized using GIWAXD and reported in our previous paper[5]. Taking VCD and TEM results into account, *at*-PMAPOSS with (*R*) or

(*S*)-BN formed helix-like structure but they are not controlled to preferred-handed helical conformation.

2. Supporting Table

Table 1. Primary structure of *at*- and *it*-PMAPOSS

Polymer	M_n[a] (g/mol)	PDI[a]	Tacticity[b]		
			mm	*mr*	*rr*
at-PMAPOSS$_{16}$	15,000	1.08	0	65	35
it -PMAPOSS$_{23}$	22,000	1.17	98	2	0
it -PMAPOSS$_{33}$	31,300	1.16	99	1	0
it -PMAPOSS$_{40}$	37,300	1.11	99	1	0

[a] Determined by SEC and [b] Determined by ^{13}C NMR.

3. Supporting Figures

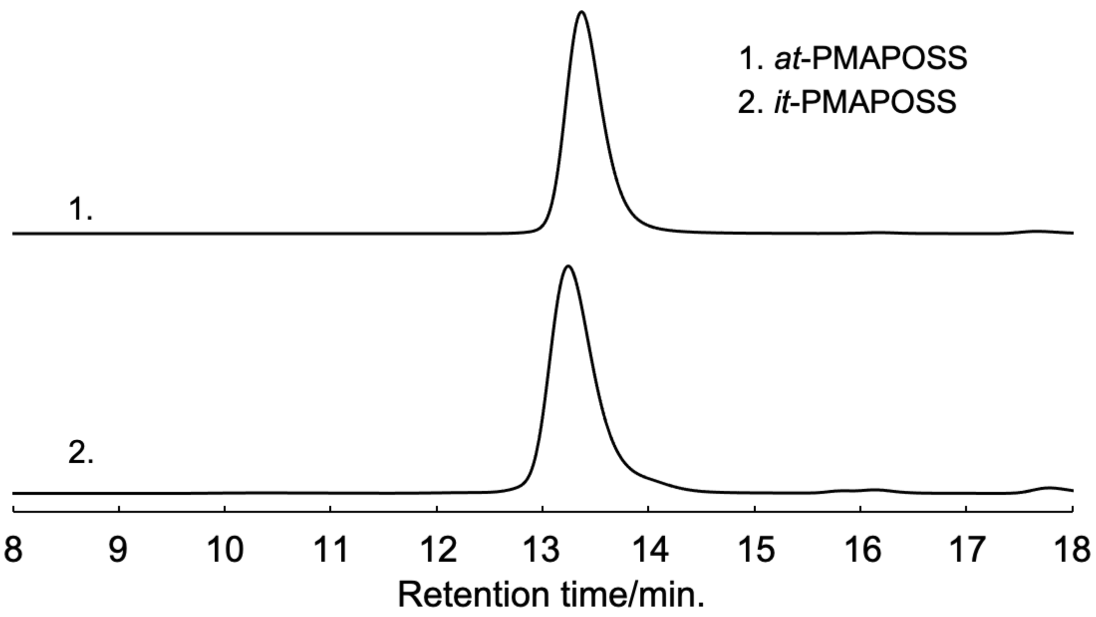

Figure S1. SEC curves of *at*-PMAPOSS and *it*-PMAPOSS.

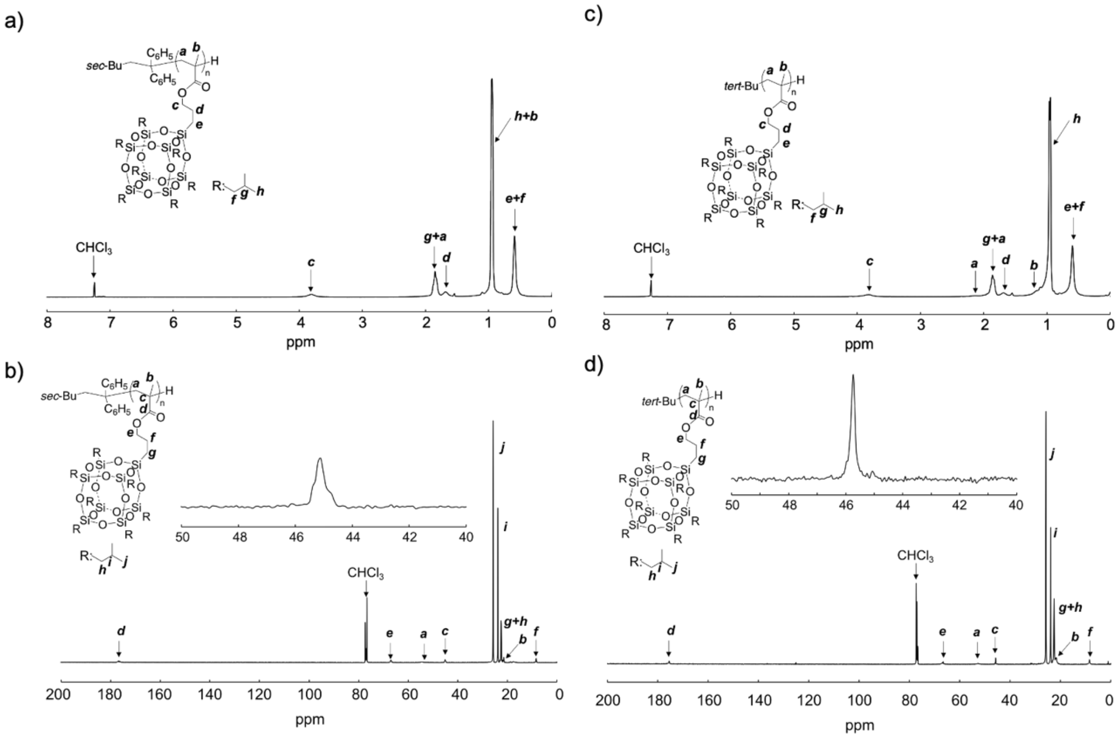

Figure S2. a) ^{1}H and b) ^{13}C NMR spectra of *at*-PMAPOSS and c) ^{1}H and d) ^{13}C NMR spectra of *it*-PMAPOSS.

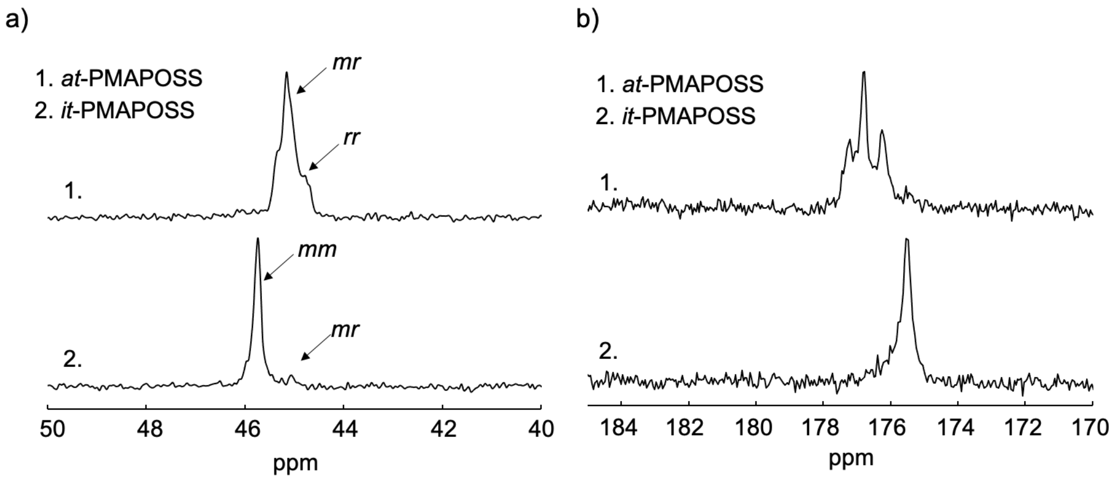

Figure S3. Highly magnified ^{13}C NMR spectra of *at*-PMAPOSS and *it*-PMAPOSS around a) 45 ppm and b) 176 ppm.

a)

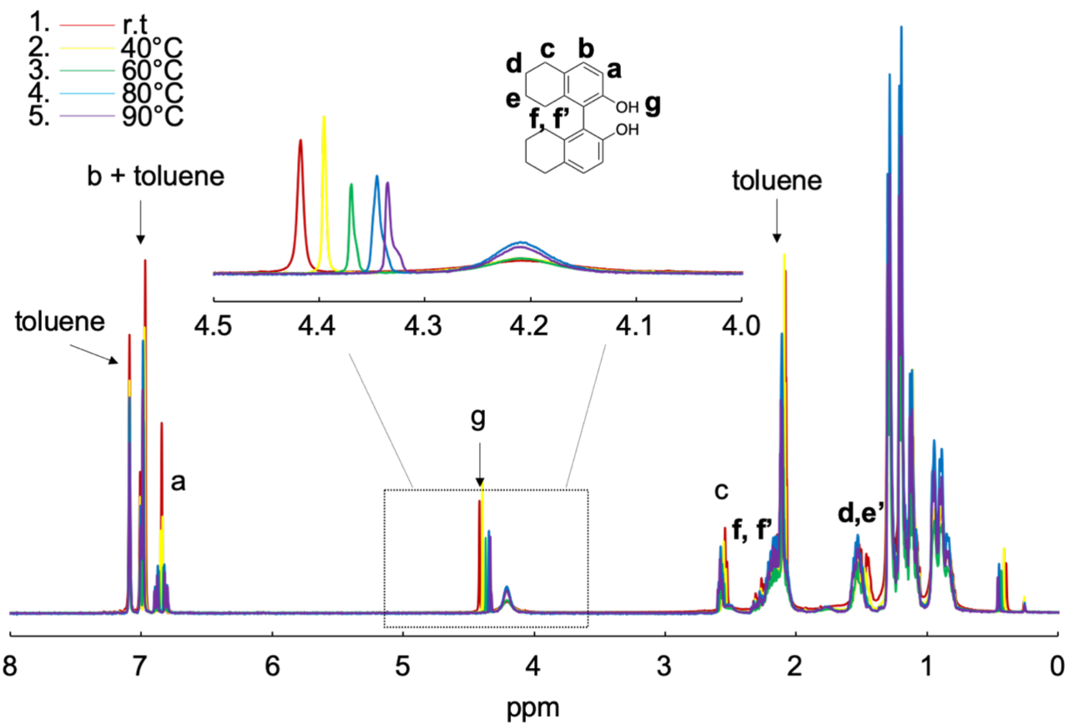

b)

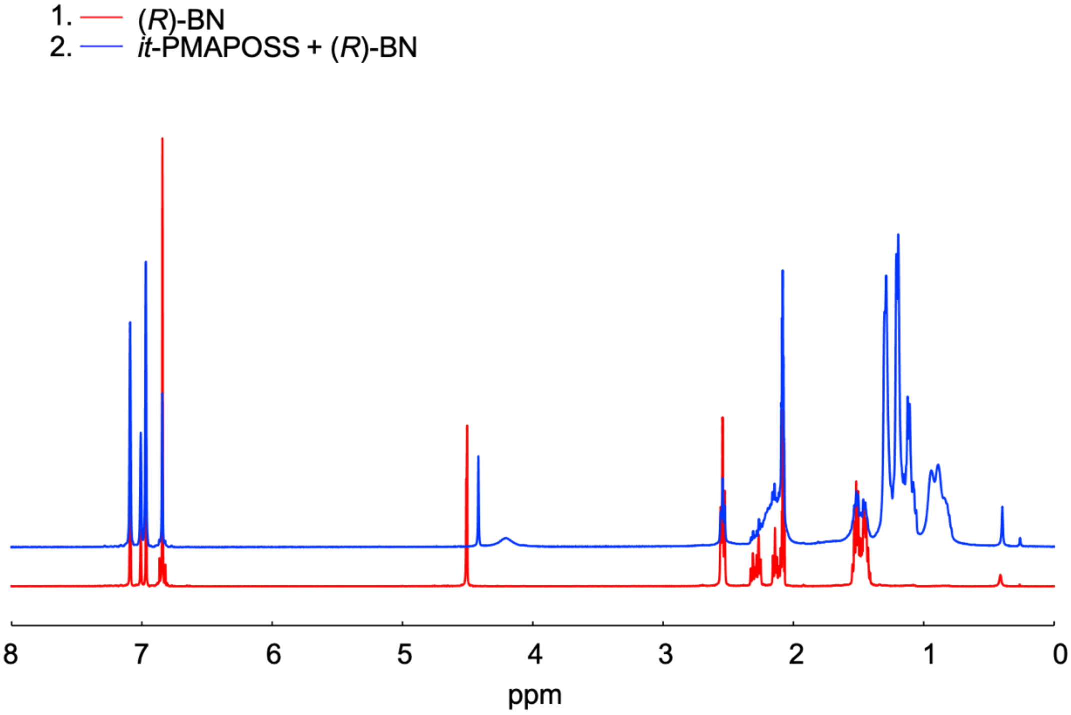

Figure S4. a) Temperature dependence of ^{1}H NMR spectra of *it*-PMAPOSS with (*R*)-BN. b) ^{1}H NMR spectra of (*R*)-BN and *it*-PMAPOSS with (*R*)-BN.

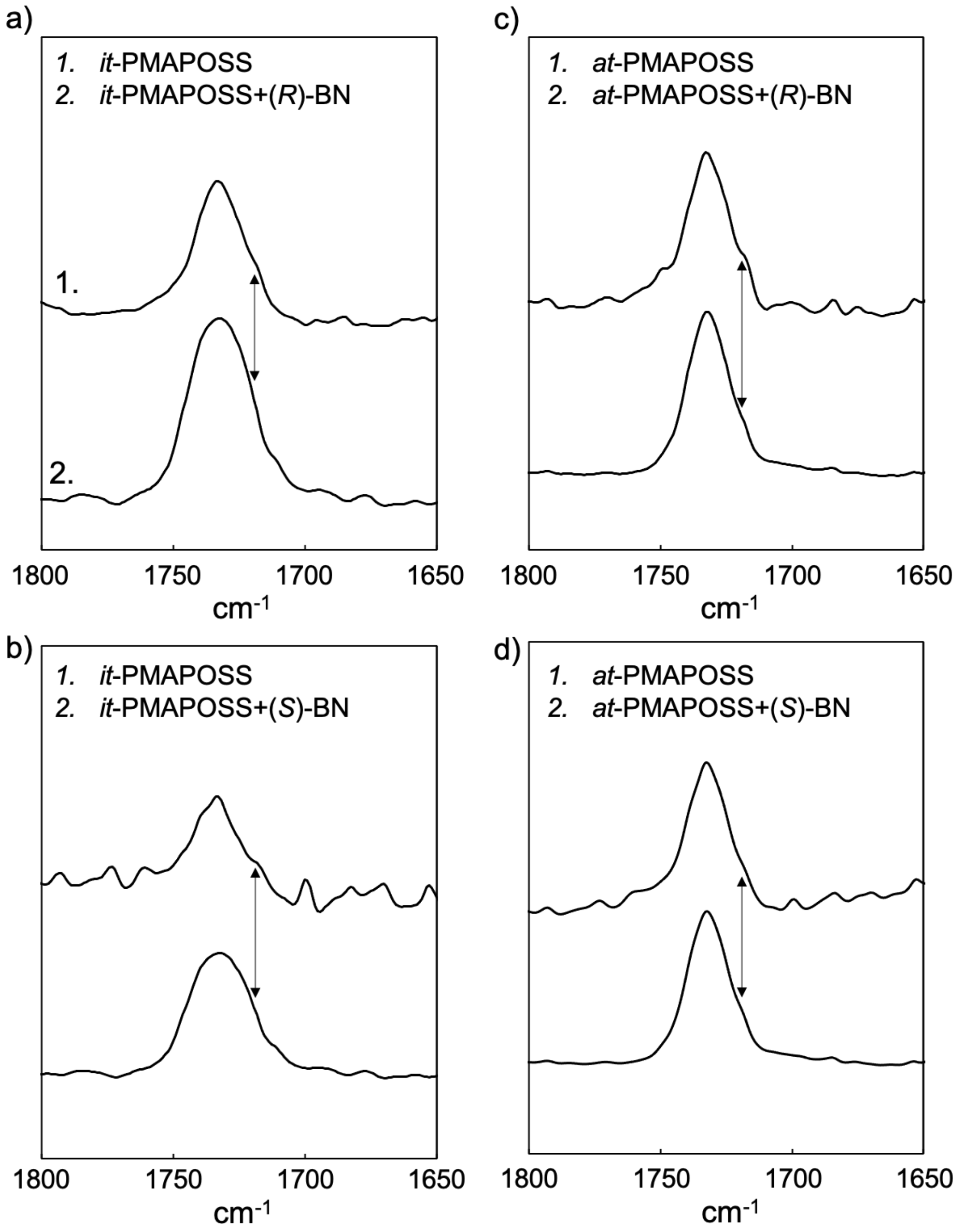

Figure S5. FT-IR spectra of *it*-PMAPOSS (a and b) and *at*-PMAPOSS (c and d) with BN.

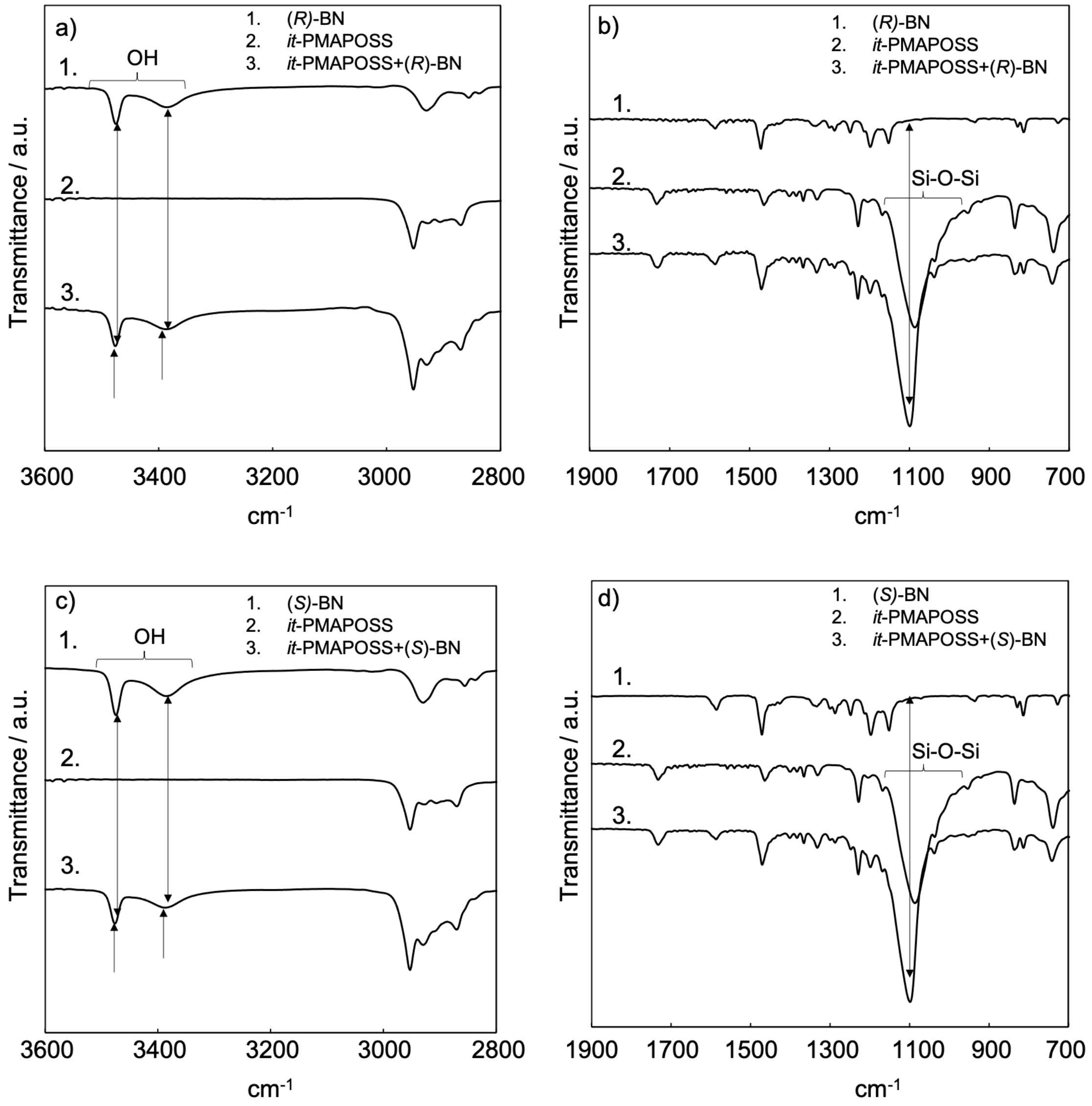
a)
1. (R)-BN
2. it-PMAPOSS
3. it-PMAPOSS+(R)-BN
OH
1.
2.
3.
Transmittance / a.u.
3600
3400
3200
3000
2800
cm-1
b)
1. (R)-BN
2. it-PMAPOSS
3. it-PMAPOSS+(R)-BN
Si-O-Si
1.
2.
3.
Transmittance / a.u.
1900
1700
1500
1300
1100
900
700
cm-1
c)
1. (S)-BN
2. it-PMAPOSS
3. it-PMAPOSS+(S)-BN
OH
1.
2.
3.
Transmittance / a.u.
3600
3400
3200
3000
2800
cm-1
d)
1. (S)-BN
2. it-PMAPOSS
3. it-PMAPOSS+(S)-BN
Si-O-Si
1.
2.
3.
Transmittance / a.u.
1900
1700
1500
1300
1100
900
700
cm-1

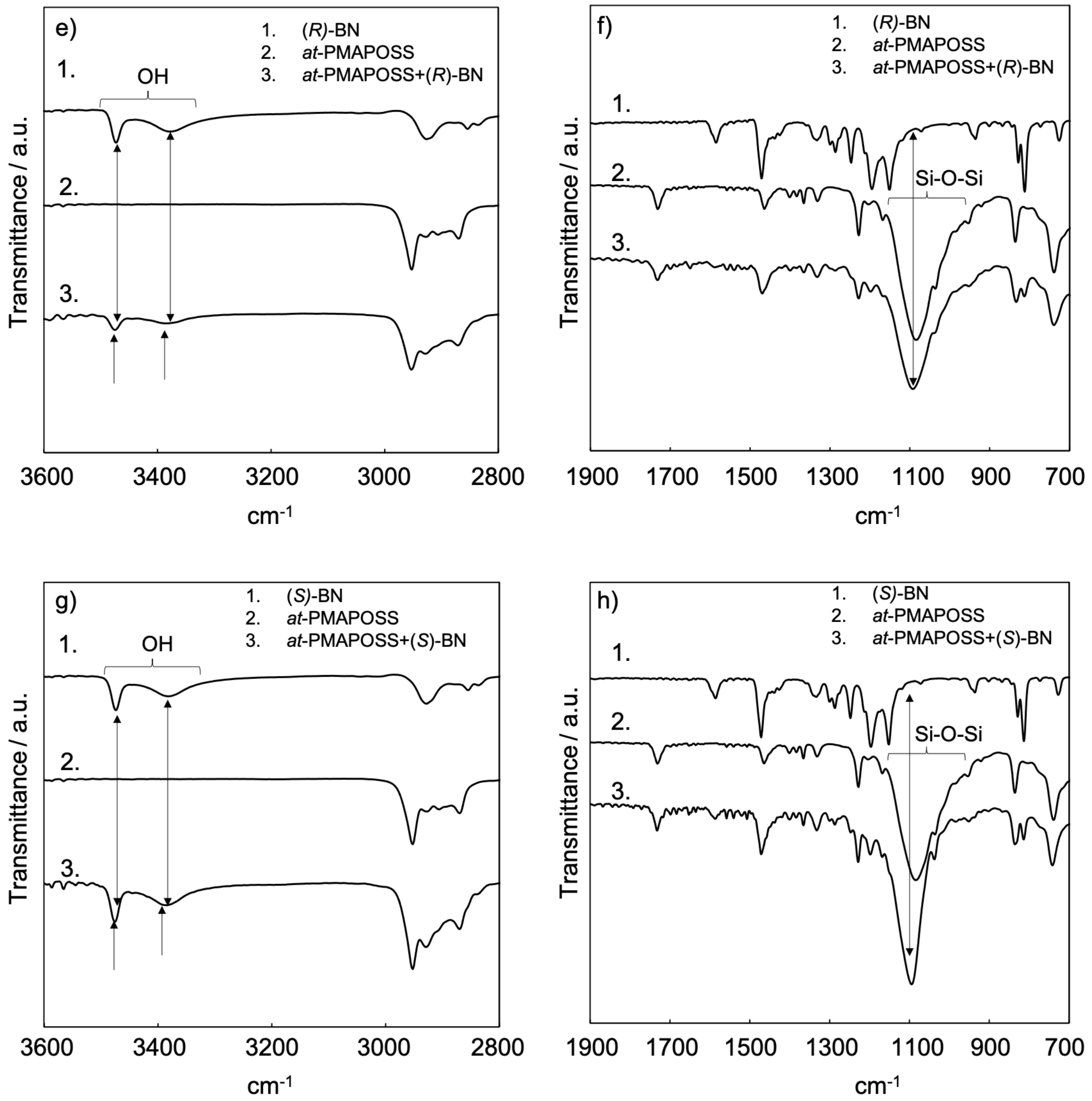

Figure S6. ATR-IR spectra of *it*-PMAPOSS (a, b, c, and d) and *at*-PMAPOSS (e, f, g, and h) with BN.

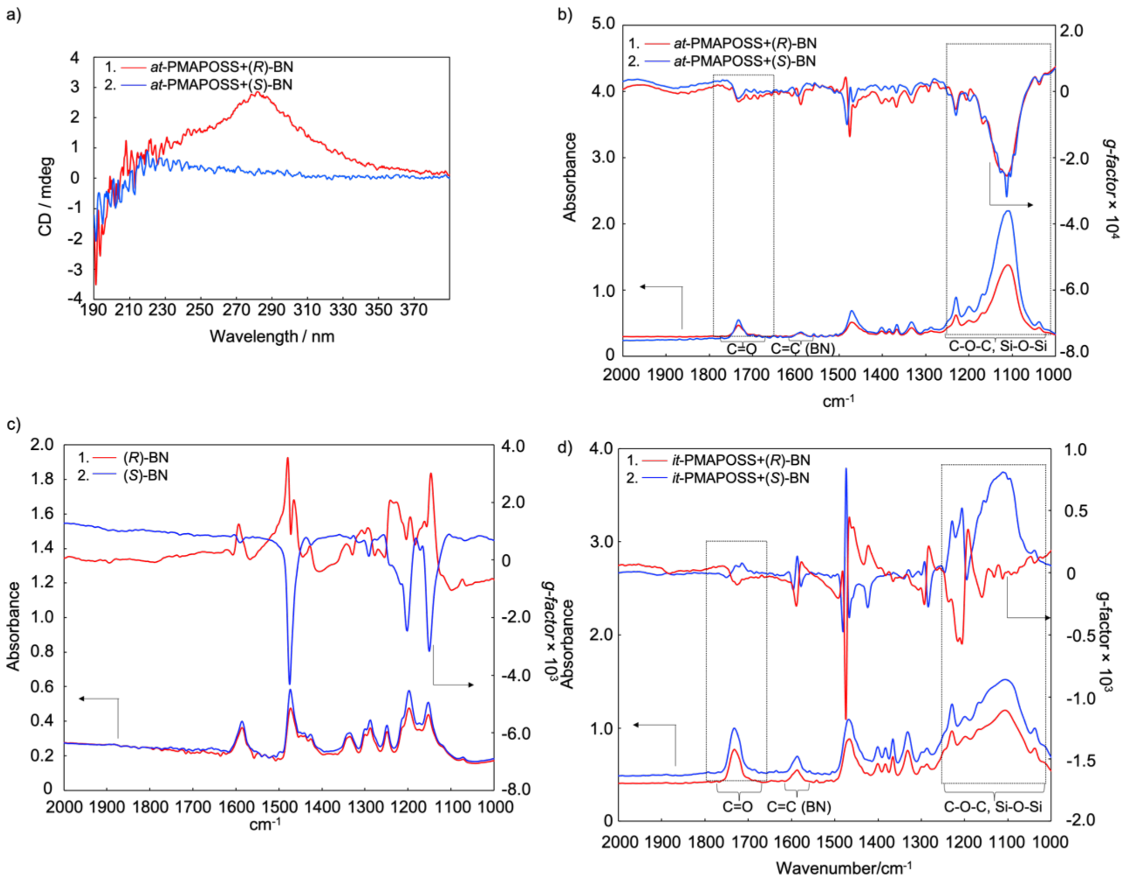

Figure S7. a) ECD and b) VCD of *at*-PMAPOSS with BN. c) VCD spectra of (*R*) and (*S*)-BN. d) VCD spectra of *it*-PMAPOSS with BN.

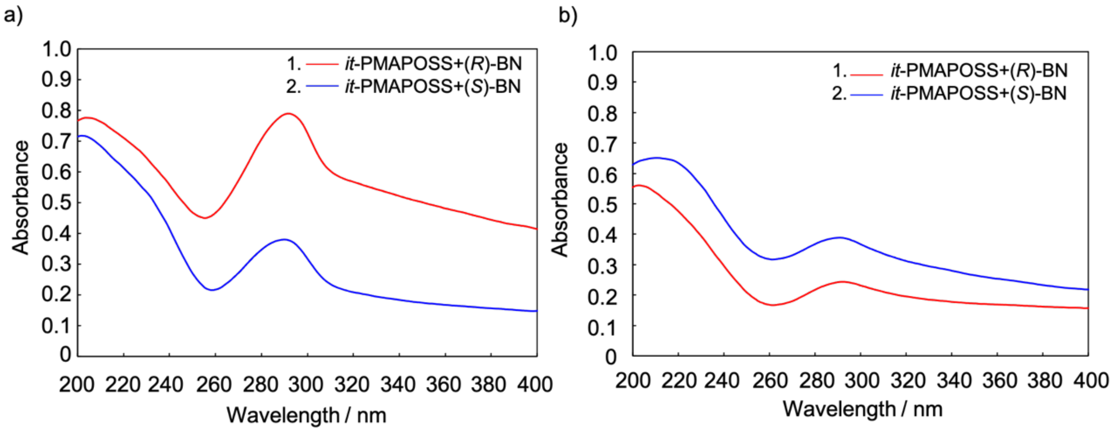

Figure S8. UV-Vis spectra of *it*-PMAPOSS with BN a) before and b) after annealing at 200°C for 30 min.

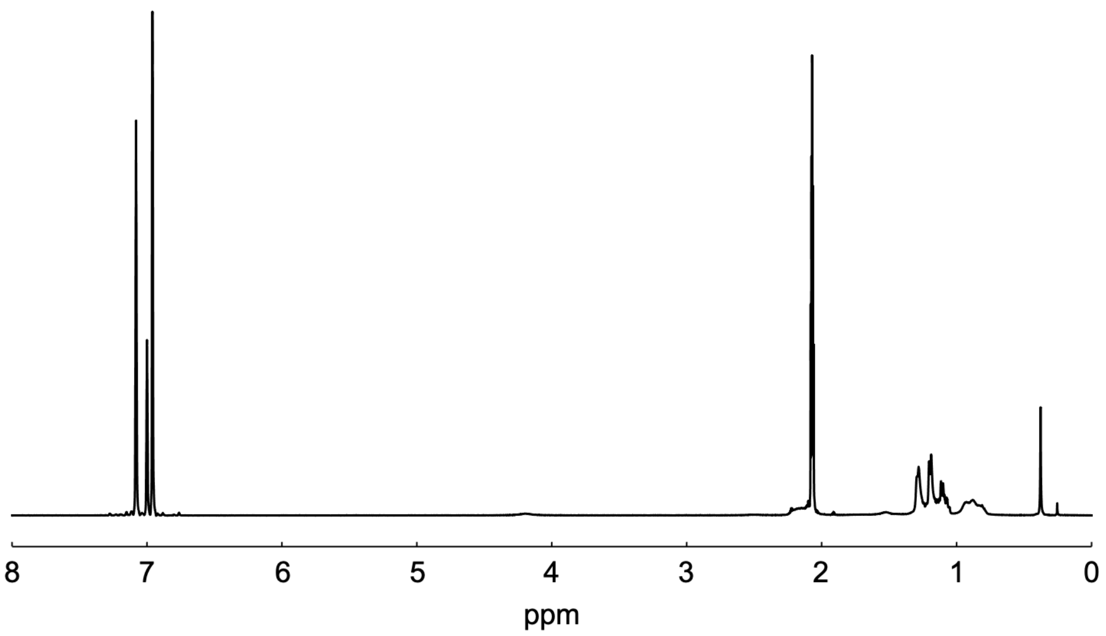

Figure S9. ^{1}H NMR spectra of *it*-PMAPOSS with BN after annealing at 200°C for 1 h.

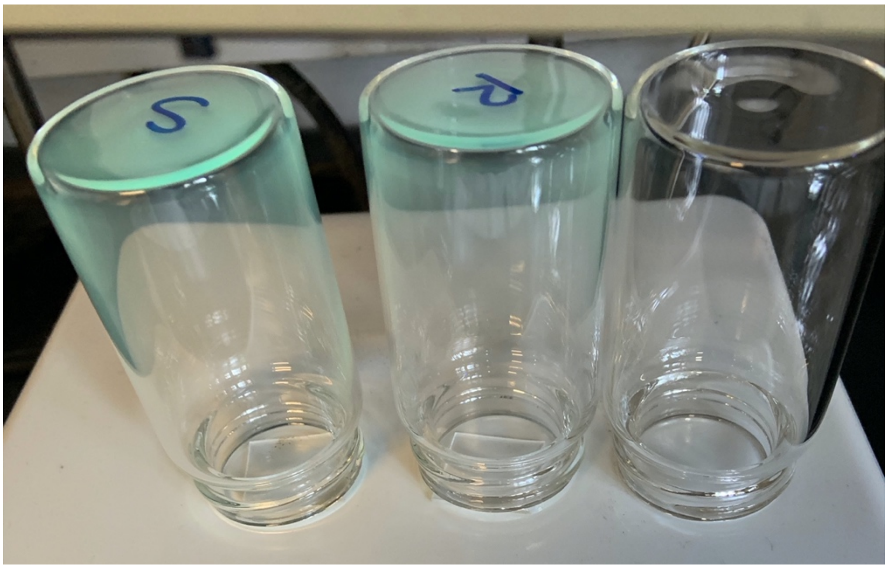

Figure S10. Photo image of thermal evaporation of (*R*)-BN and (*S*)-BN in *it*-PMAPOSS with BN. The photo image was taken under 365nm UV irradiation.

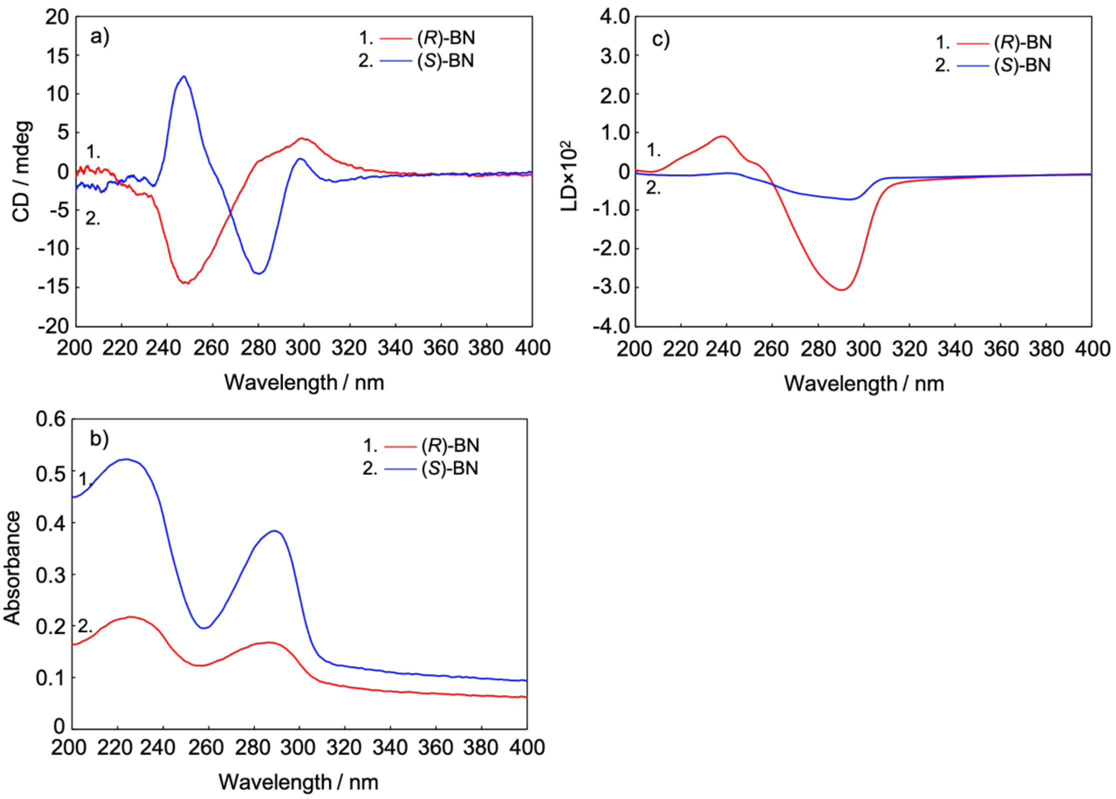

Figure S11. ECD spectra of BN film a) CD, b) UV, and c) LD.

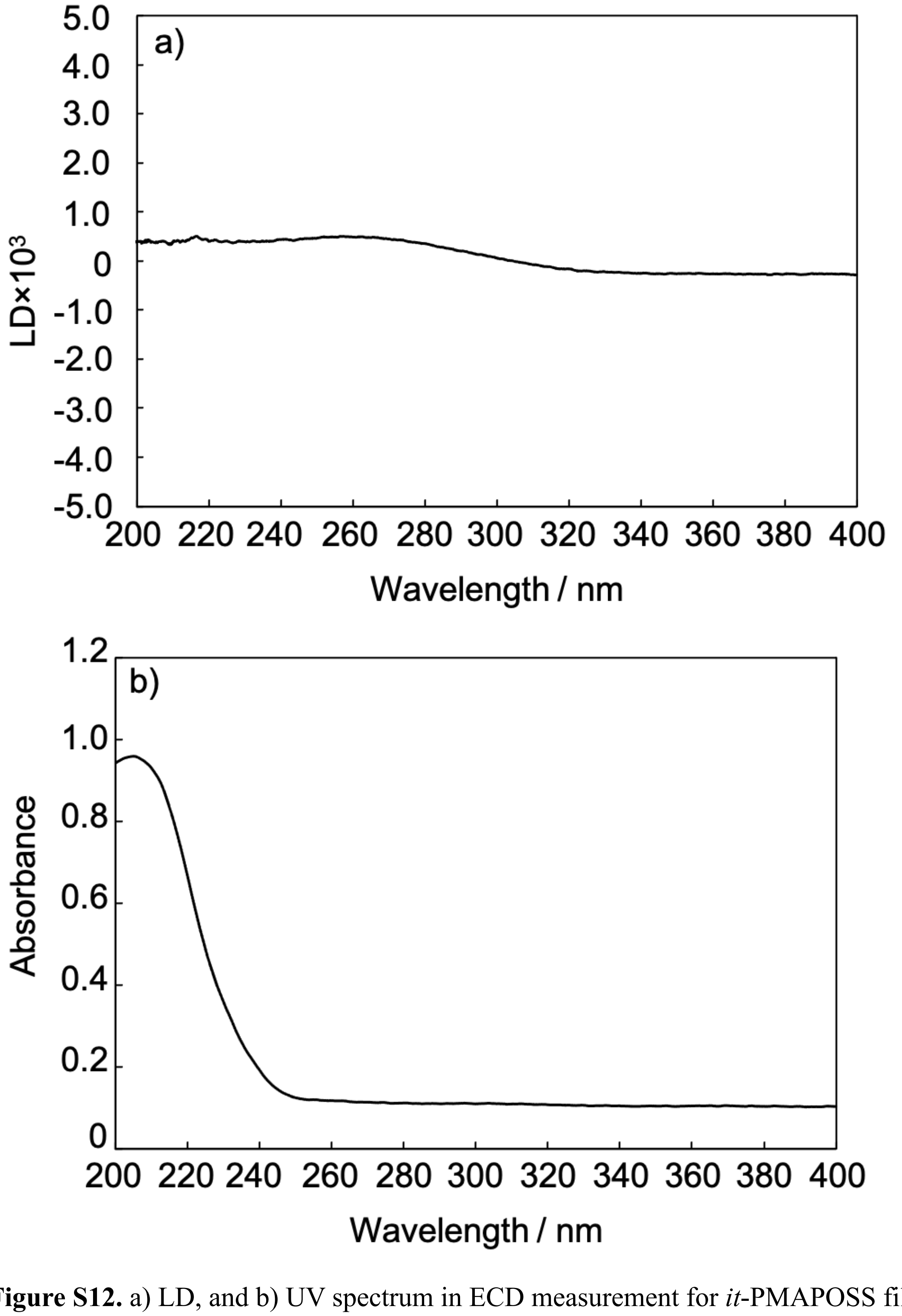

Figure S12. a) LD, and b) UV spectrum in ECD measurement for *it*-PMAPOSS film.

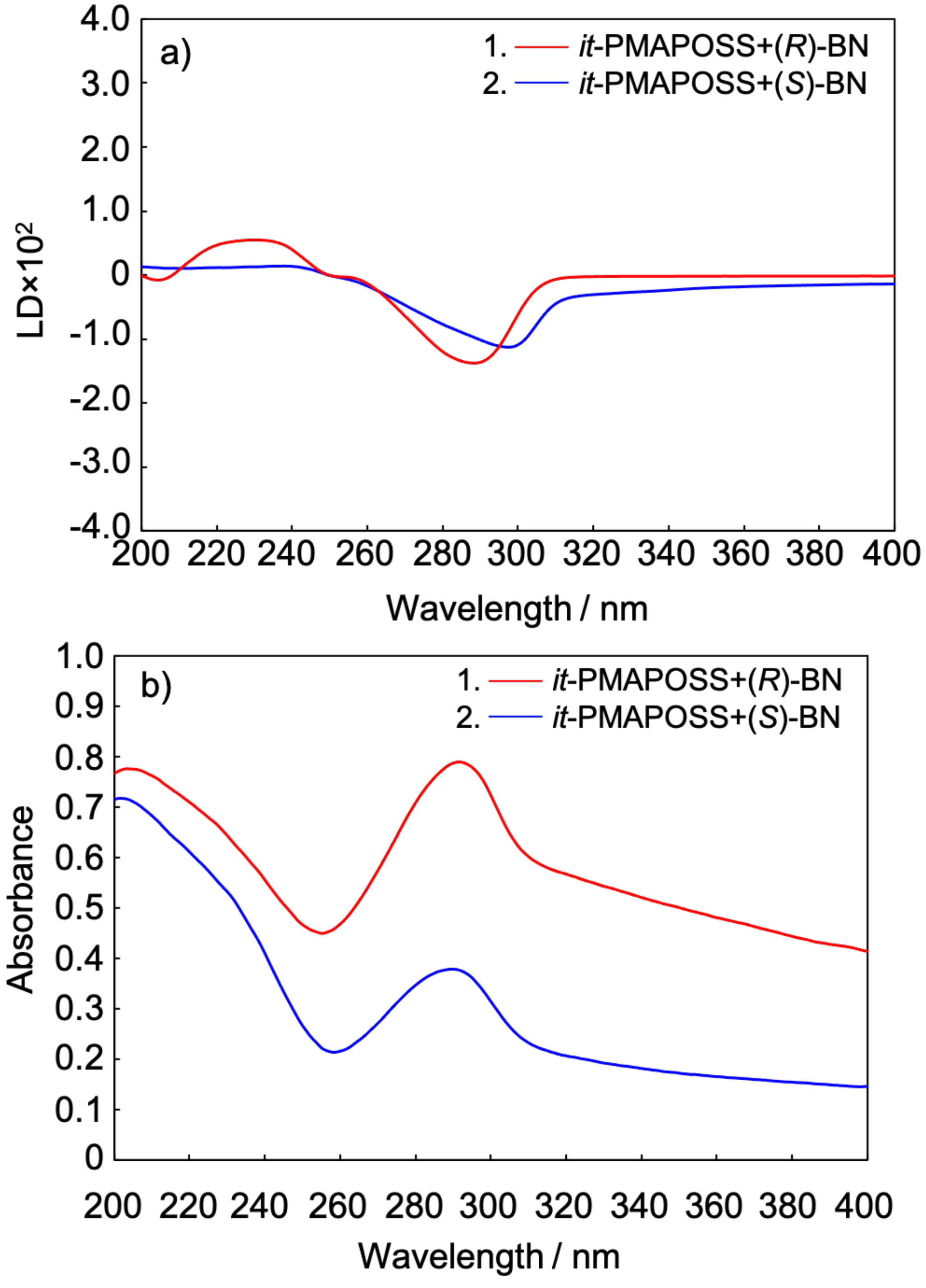

Figure S13. a) LD, and b) UV spectrum in ECD measurement for *it*-PMAPOSS with BN.

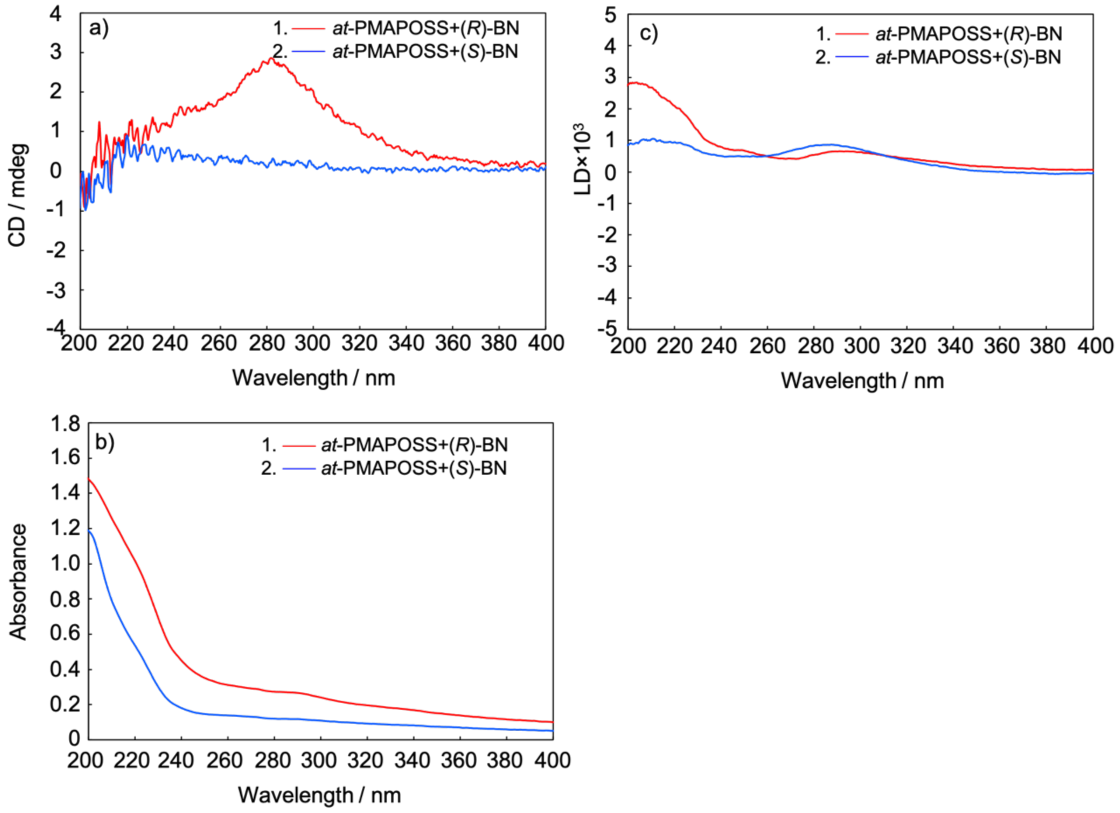

Figure S14. ECD spectra of *at*-PMAPOSS with BN a) CD, b) UV, and c) LD.

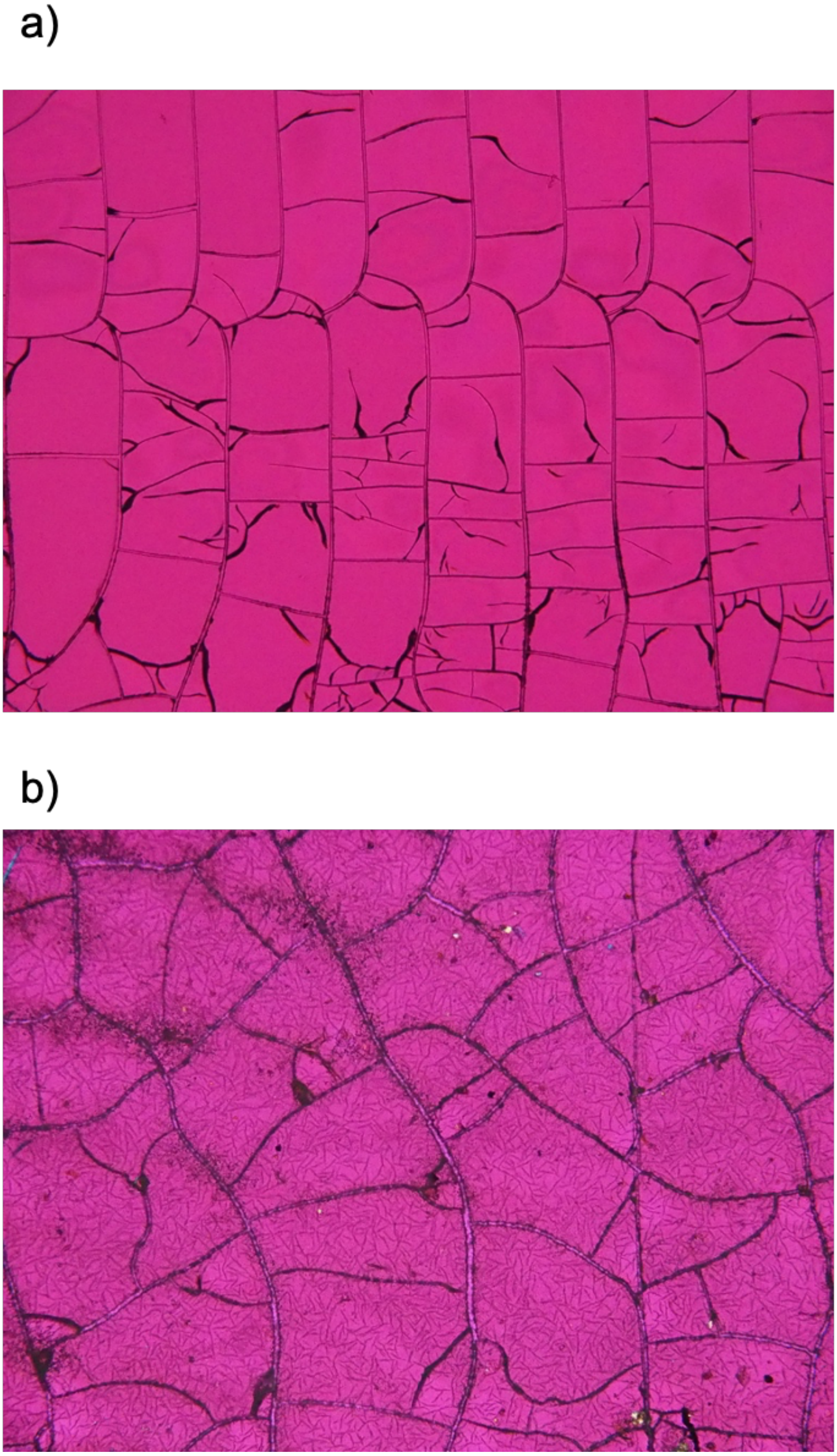

Figure S15. POM images of a) *it*-PMAPOSS and b) *it* -PMAPOSS with BN.

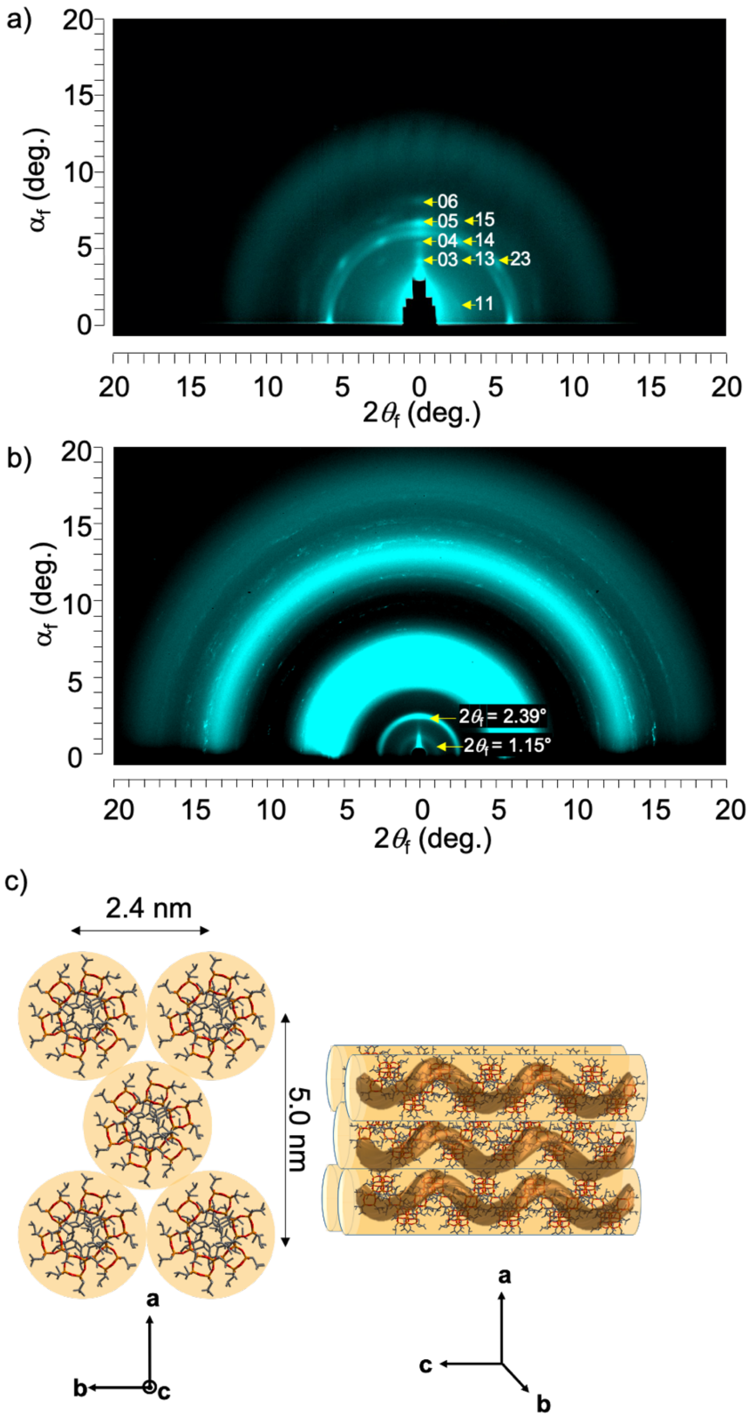

Figure S16. GIWAXD patterns of a) *it*-PMAPOSS and b) *it*-PMAPOSS doped with (*R*)- or (*S*)-BN films. c) Schematic illustrations of the ab and ac projections of the orthorhombic lattice.

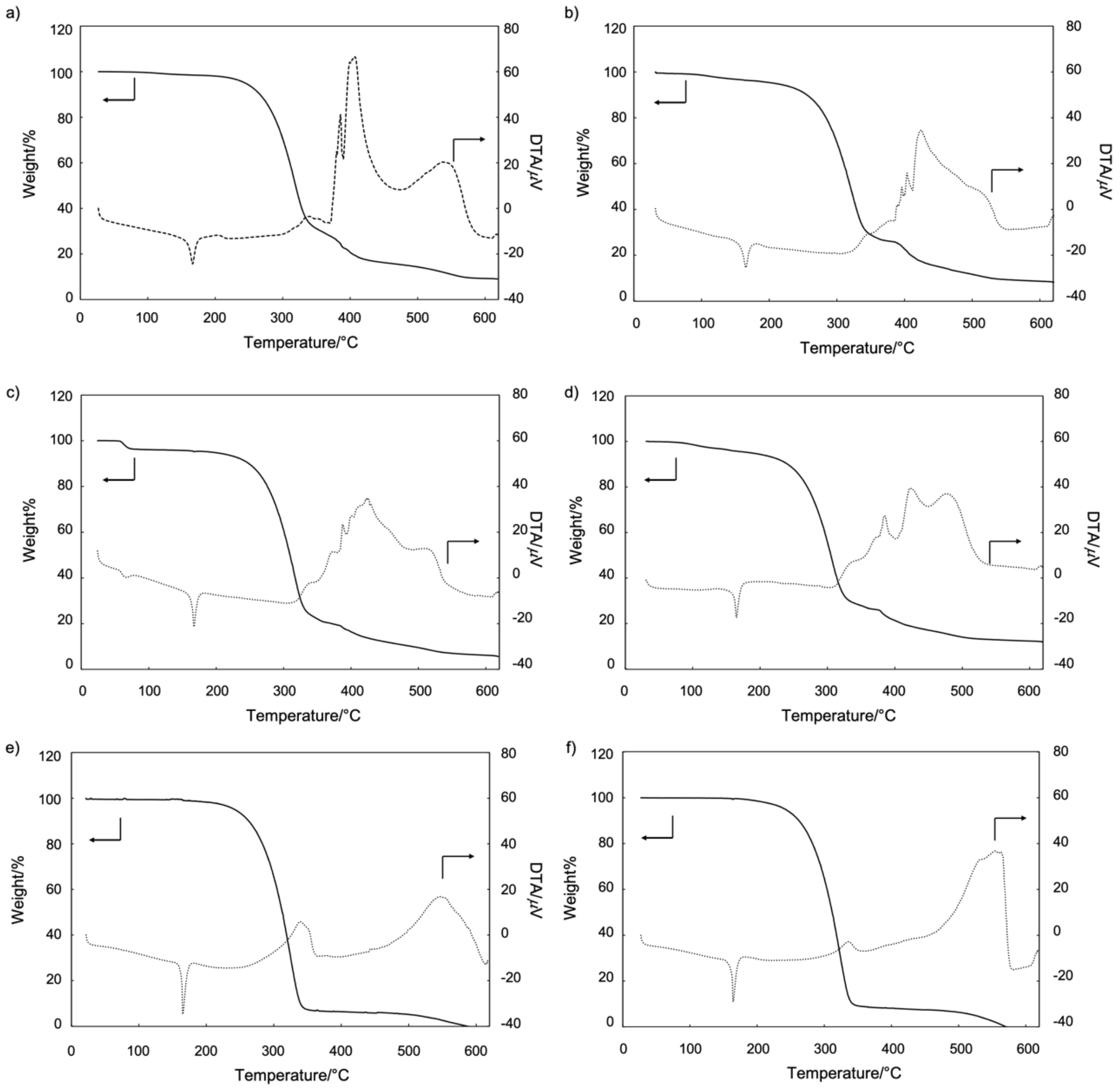

Figure S17. TGA and DTA curves for *it*-PMAPOSS with a) (*R*)-BN or b) (*S*)-BN, *at*-PMAPOSS with c) (*R*)-BN or d) (*S*)-BN, and e) (*R*)-BN or f) (*S*)-BN monomer.

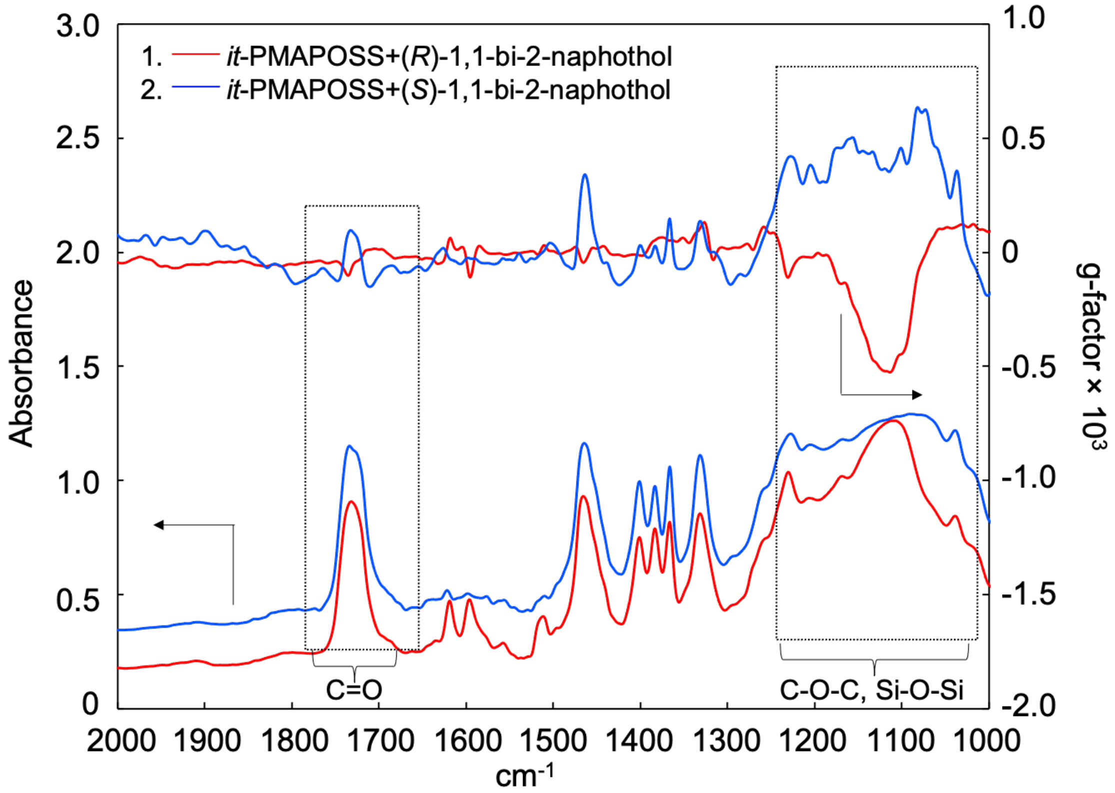

Figure S18. VCD spectra of *it*-PMAPOSS with 1,1-bi-2-naphthol.

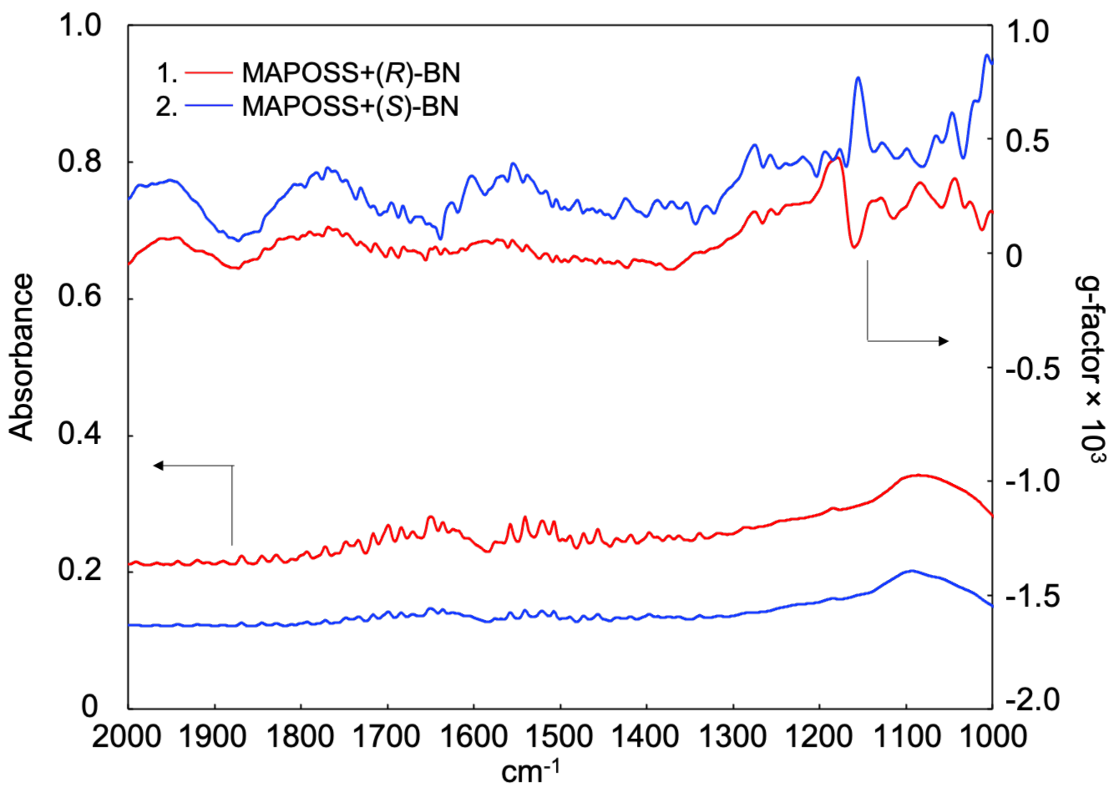

Figure S19. VCD spectra of calcinated sample prepared by MAPOSS monomer with (*R*) and (*S*)-BN.

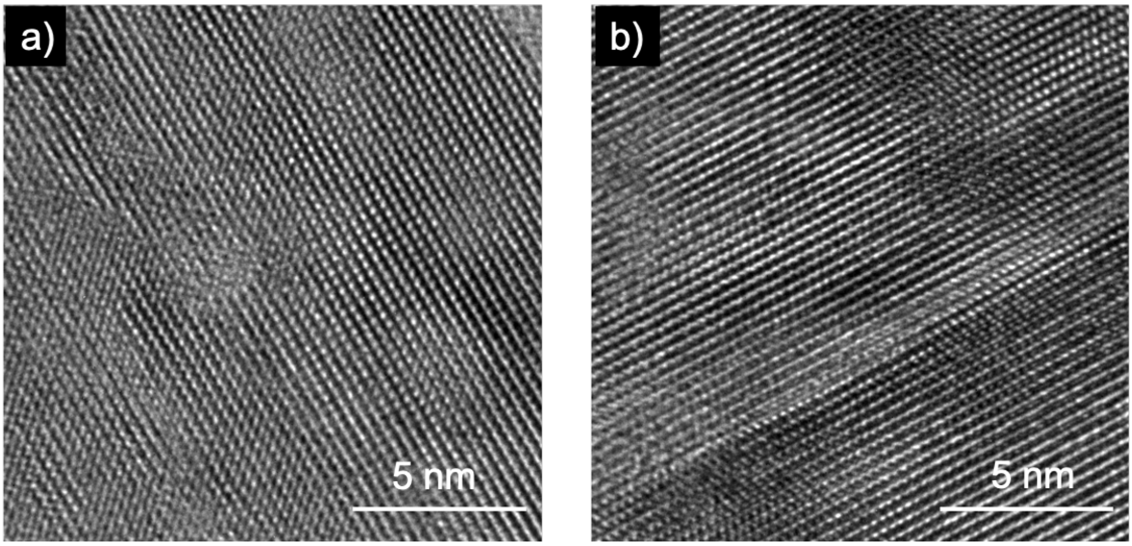

Figure S20. TEM images of the calcinated samples prepared by *at*-PMAPOSS with a) (*R*) or b) (*S*)-BN.

3. Reference

[1] T. Hirai, M. Leolukman, T. Hayakawa, M. Kakimoto, P. Gopalan, *Macromolecules* **2008**, *41*, 4558-4560.

[2] a)P. Carriere, Y. Grohens, J. Spevacek, J. Schultz, *Langmuir* **2000**, *16*, 5051-5053; b)T. Kawauchi, M. Kawauchi, T. Takeichi, *Macromolecules* **2011**, *44*, 1066-1071.

[3] G. R. Quinting, R. Cai, *Macromolecules* **1994**, *27*, 6301-6306.

[4] a)T. Suzuki, O. Yamada, Y. Murakami, Y. Takegami, Y. Watanabe, *Macromolecules* **1982**, *15*, 223-227; b)S. K. Varshney, Z. Gao, X. F. Zhong, A. Eisenberg, *Macromolecules* **1994**, *27*, 1076-1082; c)K. Hatada, T. Kitayama, K. Ute, *Prog. Polym. Sci.* **1988**, *13*, 189-276.

[5] T. Hirai, M. Leolukman, S. Jin, R. Goseki, Y. Ishida, M. Kakimoto, T. Hayakawa, M. Ree, P. Gopalan, *Macromolecules* **2009**, *42*, 8835-8843.
